# Supplementary material for: Bi-allelic variants in AP5Z1 and AP5B1 lead to retinal degeneration
Source: HGG Adv. 2026 Mar 12;7(2):100584. doi: 10.1016/j.xhgg.2026.100584 (PMC13050037; doi:10.1016/j.xhgg.2026.100584)
Supplement: Document S1. Figures S1–S7 and Table S1 [file mmc1.pdf]

HGGA, Volume 7

Supplemental information

Bi-allelic variants in *AP5Z1* and *AP5B1* lead to retinal degeneration

Hafiz Muhammad Jafar Hussain, Meng Wang, Paul Yang, Behnoosh Tasharrofi, Yumei Li, Rebecca Lynn Clark, Emma Fale-Olsen, Grace Waldow, Mohammad Keramatipour, Mostafa Asadollahi, Mark E. Pennesi, and Rui Chen

Table S1. ***In-silico*** **prediction of identified variants**

**Variant**	**Tool**	**Scores**	**Prediction**
NM_014855(AP5Z1):c.67A>T (p.Lys 23Ter)	CADD	35	deleterious
NM_014855(AP5Z1):c.928C>T (p.Arg310Ter)	CADD	45	deleterious
NM_014855: (*AP5Z1*):c.1427T>G (p.Leu476Trp)	REVEL	0.6	uncertain
	AlphaMissense	0.763	deleterious
	CADD	24.6	deleterious
	SIFT	0.001	uncertain
NM_014855(AP5Z1):c.1766C>A (p.Ser589Ter)	CADD	40	deleterious
NM_138368(*AP5B1*):c.2354T>C:(p.Leu 785Pro)	REVEL	NA	NA
	AlphaMissense	0.707	uncertain
	CADD	24.6	deleterious
	SIFT	0	deleterious

NA, not available.

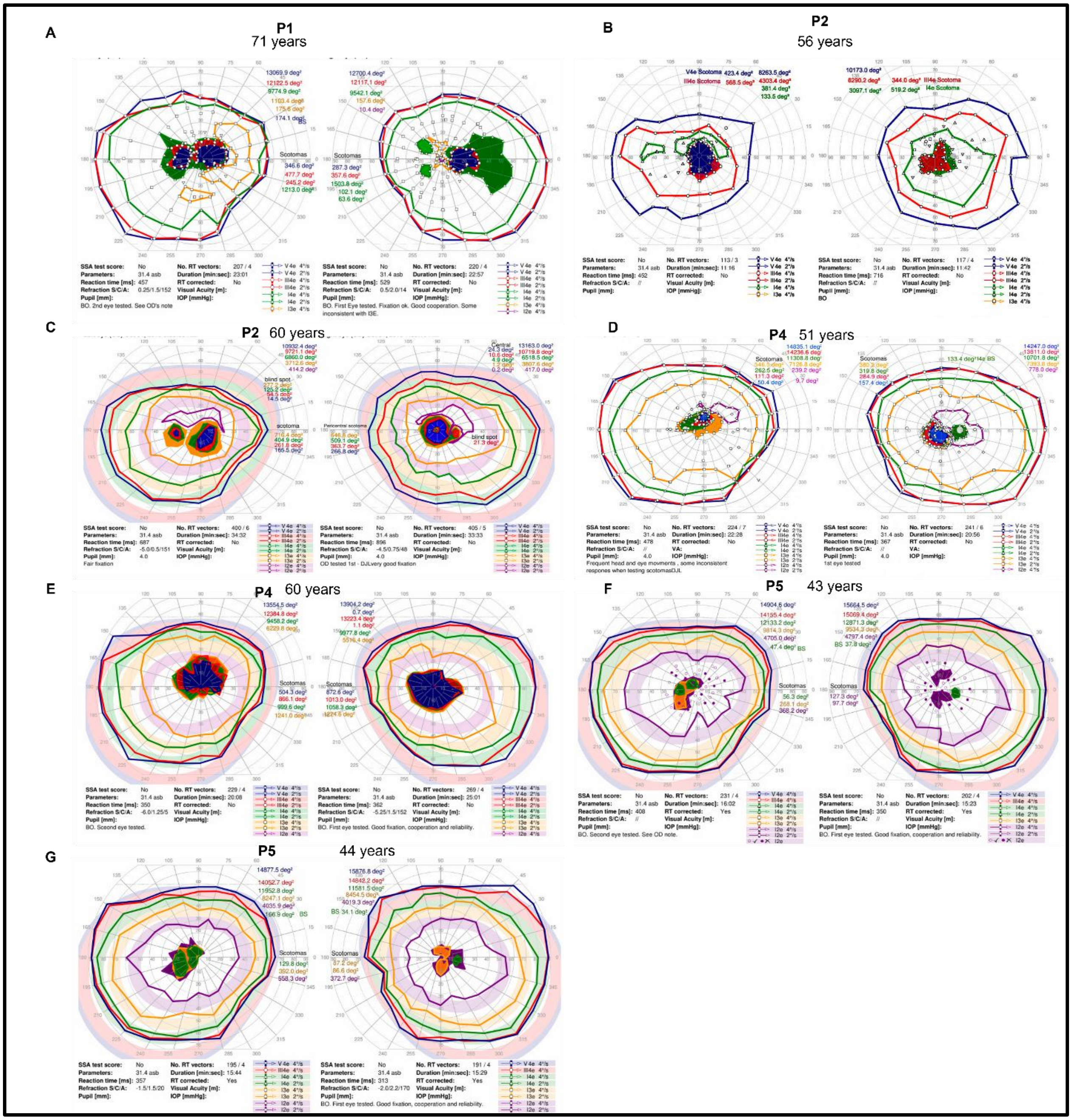

Figure S1. Kinetic Visual Fields (KVF) of P1, P2, P4 and P5. (A) KVF of patient P1 at the age of 71 years. (B) KVF of patient P2 at the age of 56 years. (C) KVF of patient P2 at the age of 60 years. (D) KVF of patient P4 at the age of 51 years. (E) KVF of patient P4 at the age of 60 years. (F) KVF of patient P5 at the age of 43 years. (G) KVF of patient P5 at the age of 44 years. All visual fields show central scotomas with preserved peripheral vision, and the findings demonstrate age-related progression, confirming chorioretinal atrophy in all patients.

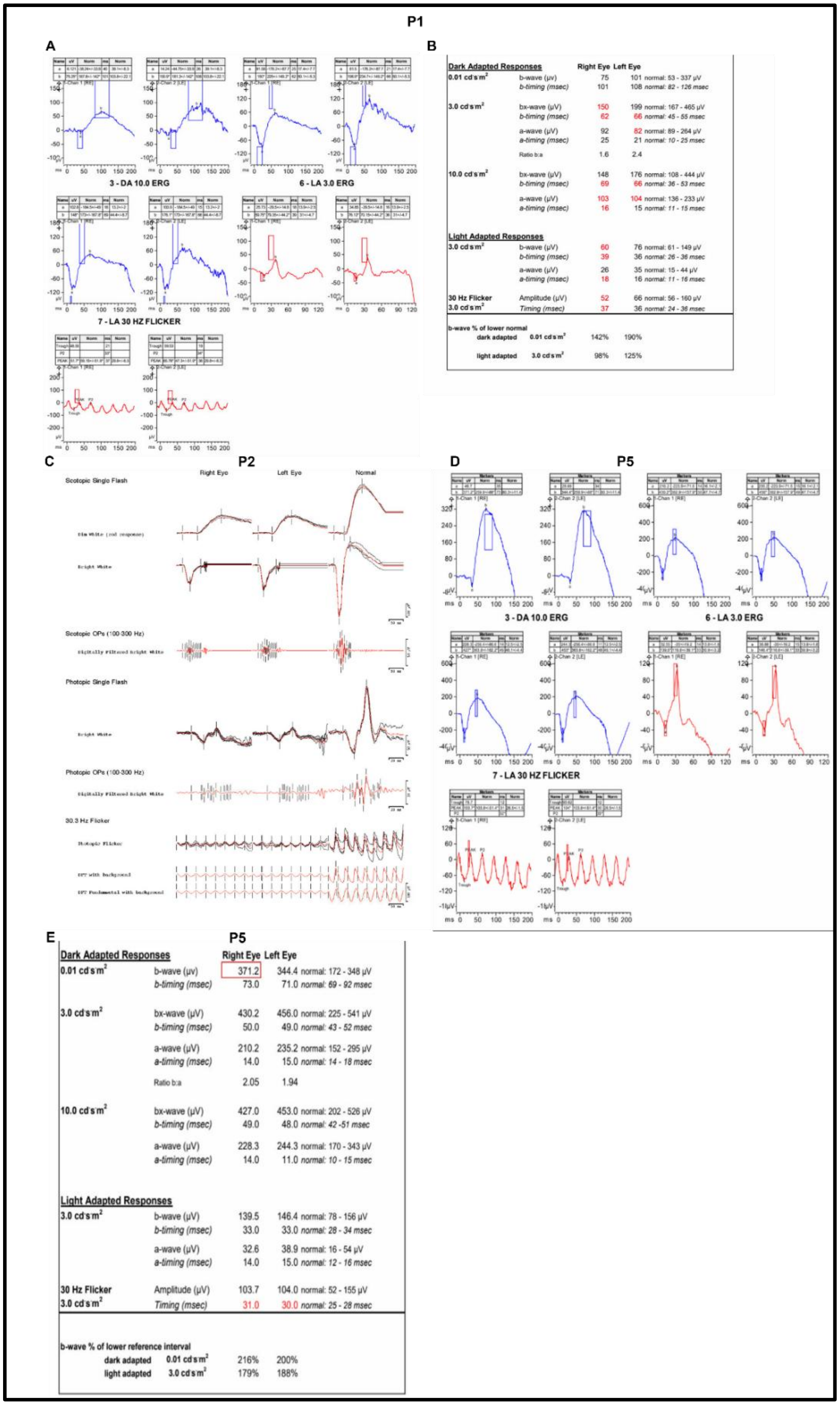

Figure S2. Full field electroretinography (ffERG) of patients P1 and P5. (A) ffERG of P1. The labeling follows standard conventions, indicating the eye's adaptive state [dark-

adapted (DA) or light-adapted (LA)] and the flash intensity (in cd·s/m²). This ERG was performed on an Espion E3 system that was calibrated to the ISCEV standards. (B) Peak amplitudes and timings for each stimulus for patient P1. These recordings demonstrate normal rod function but show mild cone dysfunction as indicated by borderline amplitudes and prolonged timings. Thes finding are consistent with macular dysfunction. (C) ffERG of P2. (D) ffERG of P5. (E) Peak amplitudes and timings for each stimulus for patient P5. These recordings demonstrate normal rod function but show mild cone dysfunction as indicated by prolonged timings of the 30Hz flicker responses.

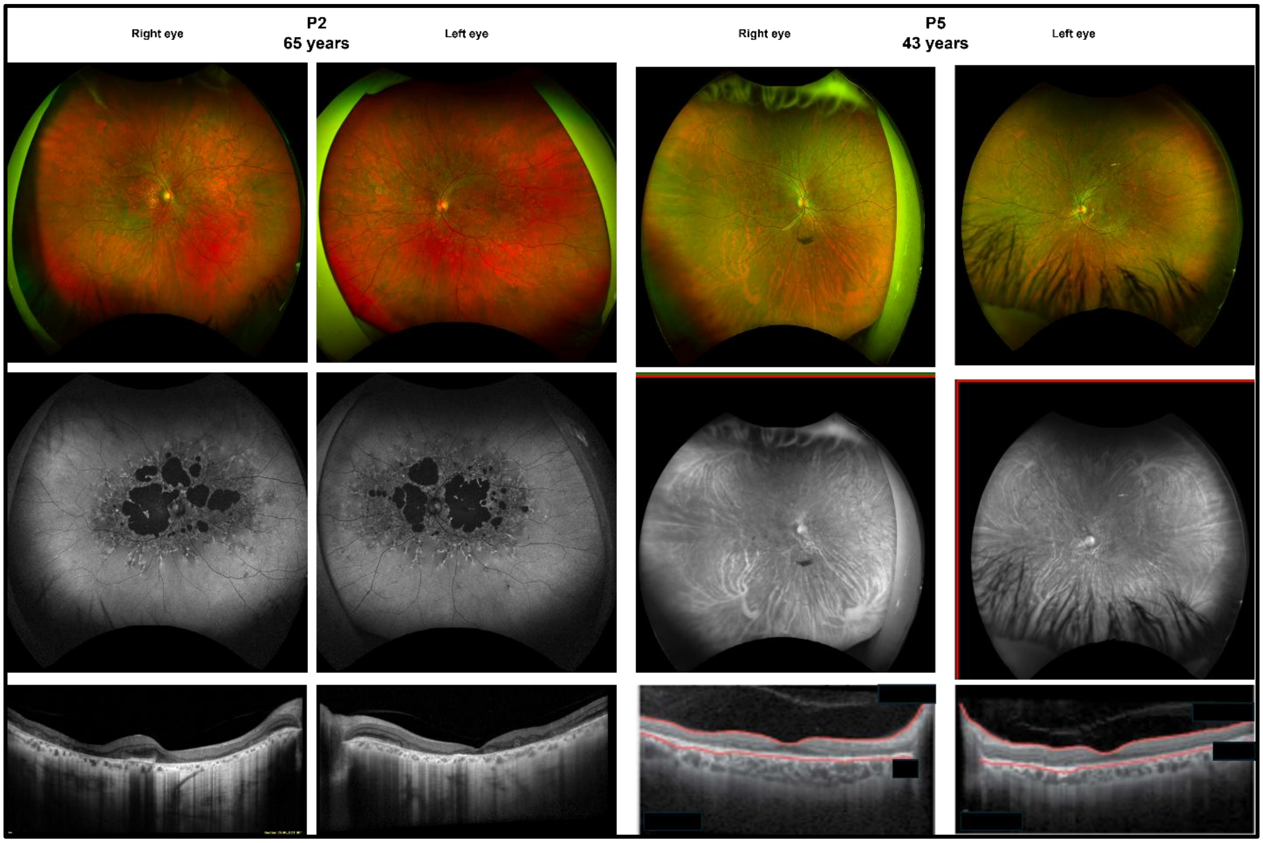

Figure S3. Multimodal retinal imaging of patients P2 and P5 carrying *AP5Z1* and *AP5B1* bi-allelic variants respectively. The top row displays multicolor or pseudocolor fundus images, the middle row shows fundus autofluorescence (FAF) images, and the bottom row presents optical coherence tomography (OCT) images in each panel.

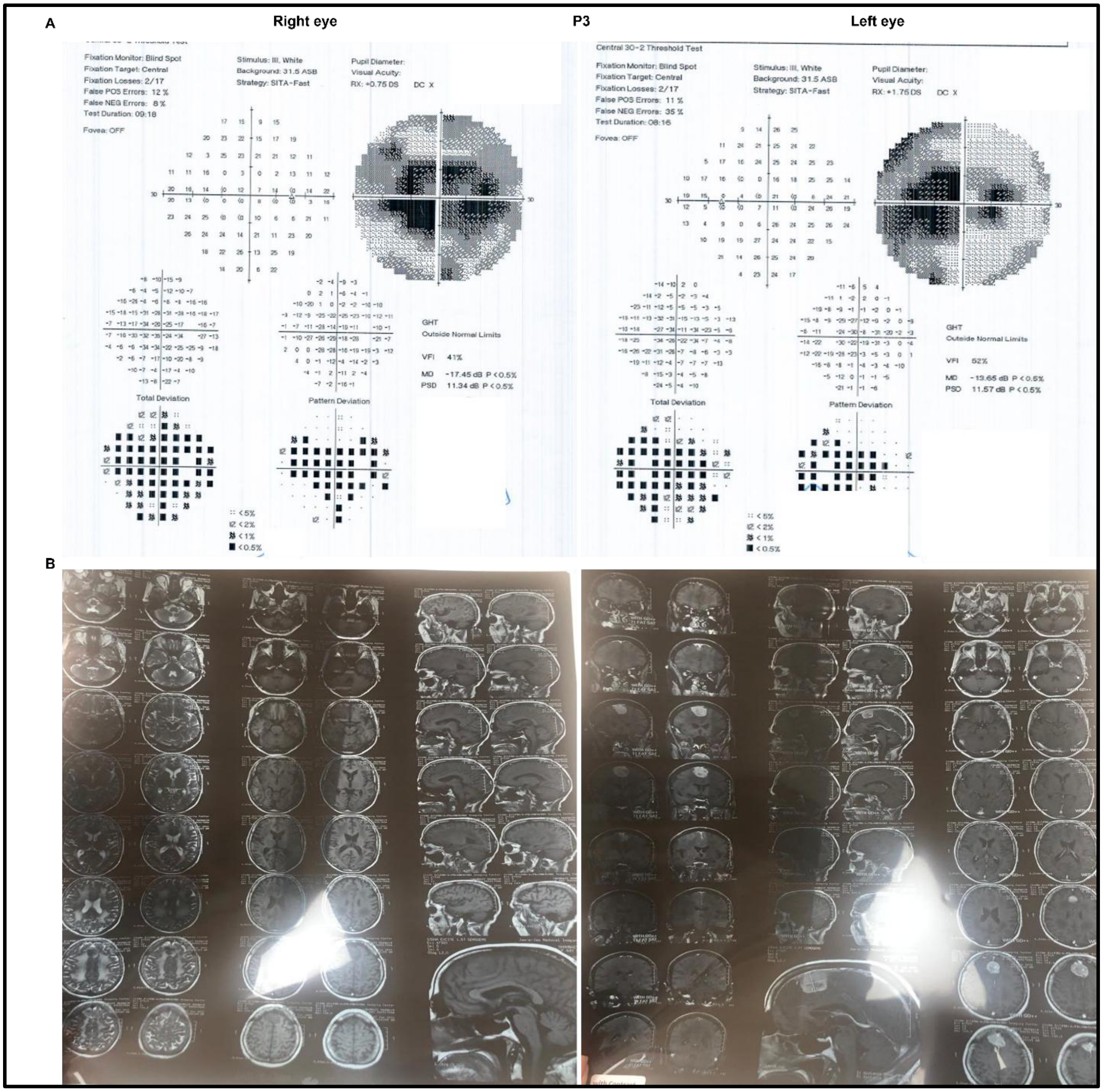

Figure S4. Humphrey Visual fields and brain MRI of patient P3. (A) Visual field test of patient P3. (B) Brain MRI of patient P3.

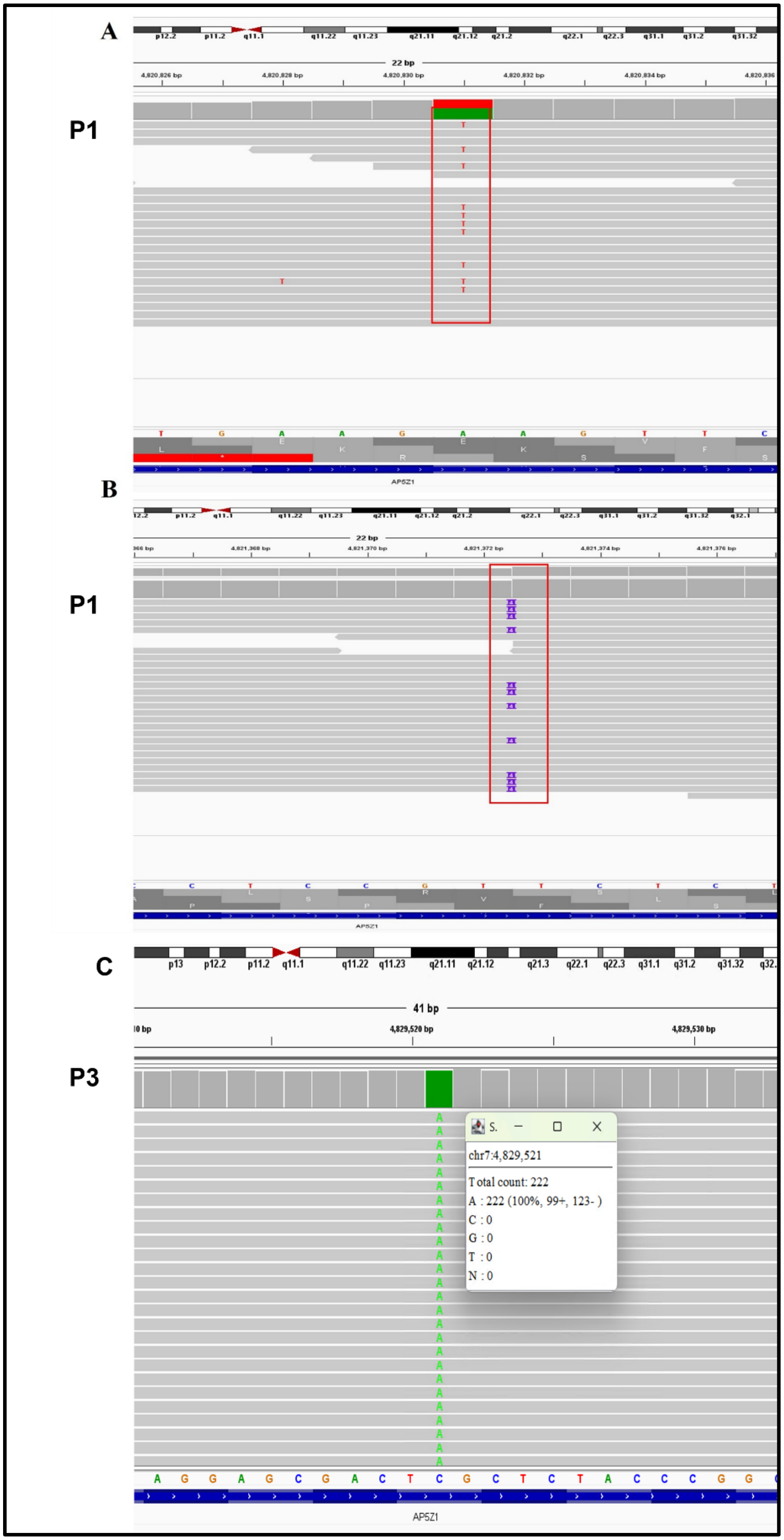
A
P1
22 bp
AP5Z1
B
P1
22 bp
AP5Z1
C
P3
41 bp
4,829,520 bp
4,829,530 bp
chr7:4,829,521
Total count: 222
A : 222 (100%, 99+, 123-)
C : 0
G : 0
T : 0
N : 0
AGGAGCGACTCGCTCTACCCGG
AP5Z1

Figure S5. IGV and identified variant's location in P1 and P3. (A) IGV plot showing the variant region of patient (P1) affected with *AP5Z1*-related retinopathy had a likely pathogenic heterozygous stop-gain chr7:4820831-A-T. (B) IGV plot showing the variant region of patient (P1) affected with *AP5Z1*-related retinopathy had a likely pathogenic heterozygous insertion of four nucleotides chr7:4821372-T-TTCTC in *AP5Z1* gene. (C) IGV plot showing the variant region of patient (P3) affected with *AP5Z1*-related retinopathy had a likely pathogenic homozygous nonsense variant chr7-4829521-C-A in *AP5Z1* gene.

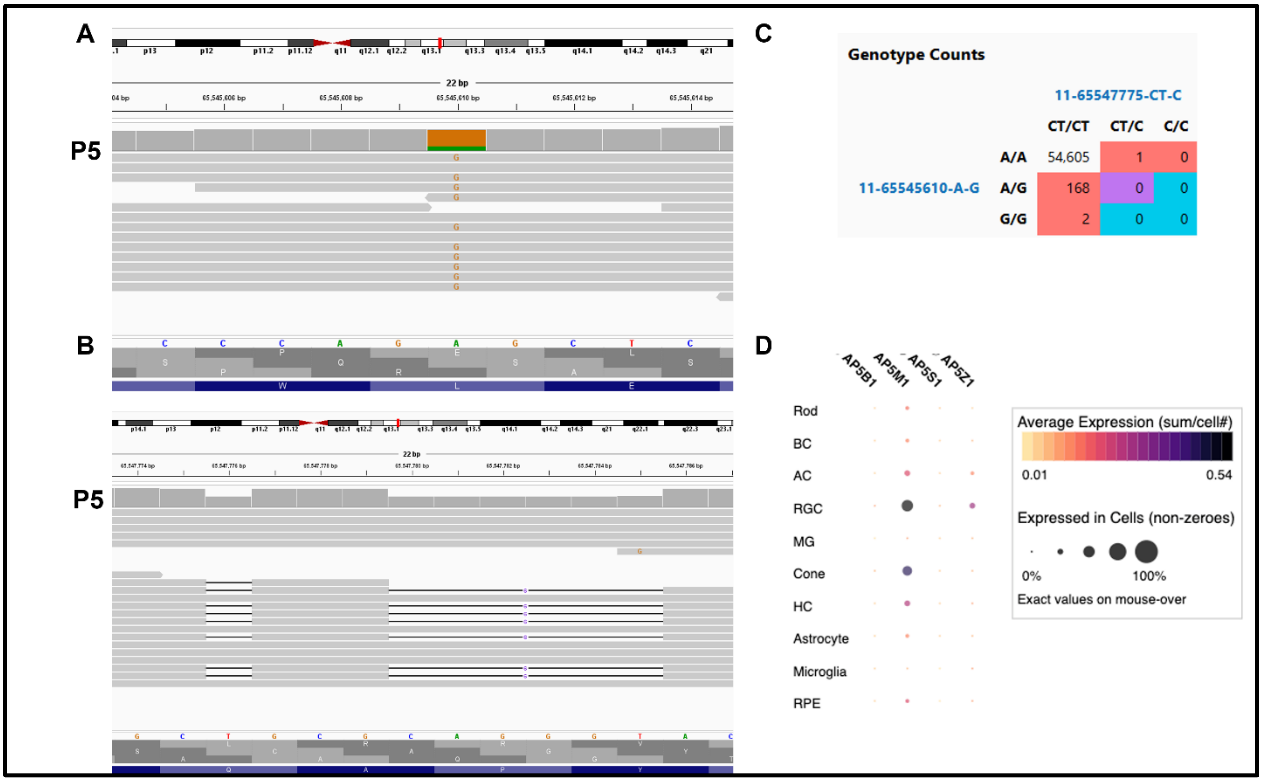

Figure S6. IGV of P5 and AP5 complex genes expression. (A) IGV plot showing the variant region of patient (P5) affected with *AP5B1*-related retinopathy had heterozygous missense variant chr11-65545610-A-G. (B) IGV plot showing the variant region of patient (P5) affected with *AP5B1*-related retinopathy had a heterozygous deletion of one nucleotide, resulting in stop codon chr11-65547775-CT-C. (C) Phasing analysis from gnomAD v2 of identified variants in patient P5 (chr11-65545610-A-G: (NM_138368) c.2354T>C:p.Leu785Pro/chr11-65547775-CT-C: c.188delA:p.Gln63Argfs*95. (D) Single nuclei expression of *AP5* complex genes in major retina cell classes.

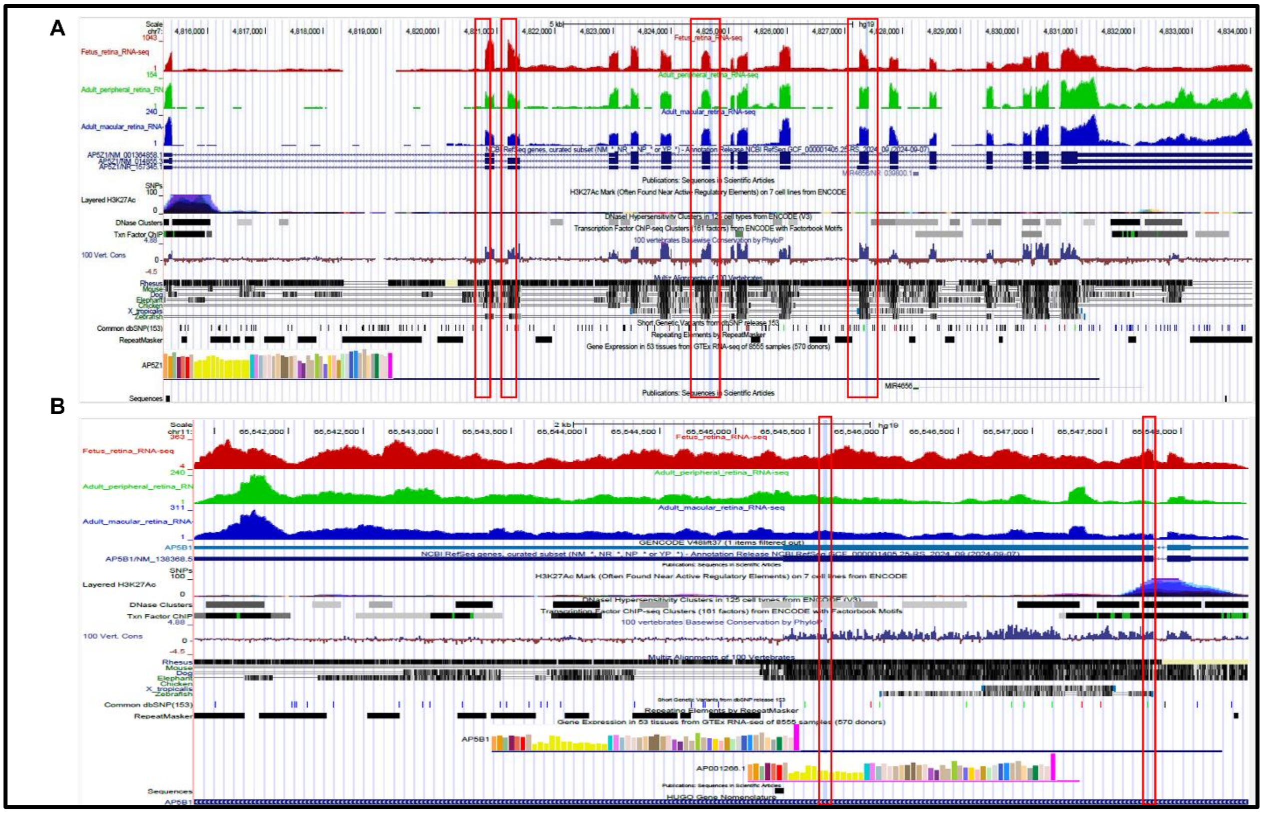

Figure S7. RNA-seq expression of AP5Z1 (A) and AP5B1 (B) in fetus and adult retina and location of identified variants in both genes (red enclosed).
