# Supplementary material for: Bi-allelic variants in AP5Z1 and AP5B1 lead to retinal degeneration
Source: HGG Adv. 2026 Mar 12;7(2):100584. doi: 10.1016/j.xhgg.2026.100584 (PMC13050037; doi:10.1016/j.xhgg.2026.100584)
Supplement: Document S2. Article plus supplemental information [file mmc2.pdf]

HGG Advances

ARTICLE

Bi-allelic variants in *AP5Z1* and *AP5B1* lead to retinal degeneration

Hafiz Muhammad Jafar Hussain,[1] Meng Wang,[2] Paul Yang,[3] Behnoosh Tasharrofi,[4] Yumei Li,[2] Rebecca Lynn Clark,[3] Emma Fale-Olsen,[3] Grace Waldow,[3] Mohammad Keramatipour,[4] Mostafa Asadollahi,[4] Mark E. Pennesi,[3,5] and Rui Chen[2,6,*]

Summary

Inherited retinal diseases (IRDs) comprise a diverse group of disorders that frequently lead to progressive vision impairment and blindness. Despite advances in genetic testing, a significant number of IRD cases remain genetically unsolved, often due to unidentified disease-associated genes or variants. This study reports additional cases for the newly discovered IRD genes of the AP-5 complex. A comprehensive ophthalmological evaluation was performed for all patients, including retinal imaging (multimodal imaging), visual field testing, and electroretinogram (ERG) testing. Whole-genome and -exome sequencing (WGS and WES) were performed for clinically unsolved IRD patients, and data were analyzed to identify underlying causal variants. The identified variants were subsequently validated using Sanger sequencing. Five unrelated patients from Europe and Iran were identified with a distinctive macular degeneration associated with bi-allelic variants in *AP5Z1* (HGNC: 22197) and *AP5B1* (HGNC: 25104), subunits of the vesicular fifth adaptor protein (AP-5) complex. The AP-5 complex is the part of the intracellular trafficking machinery thought to be involved in cellular homeostasis and lysosomal functioning in the retinal pigment epithelium (RPE). The identification of bi-allelic variants in two proteins of the AP-5 complex expand the characterization of AP-5 genes in sustaining and preserving normal macular function.

Introduction

Inherited retinal diseases (IRDs) are a group of genetic disorders that cause progressive vision loss, primarily due to the degeneration or dysfunction of rod and cone photoreceptors. Although IRDs are classified as monogenic disorders, their genetic basis is complex, involving mutations in genes critical for the proper function of various retinal cells, particularly photoreceptors and the retinal pigment epithelium (RPE).[1] These conditions can present autosomal-recessive, autosomal-dominant, X-linked, or maternal (mitochondrial) inheritance patterns depending on the specific pathogenic variants involved. To date, mutations in 483 genes have been linked to IRDs (https://retigene.erdc.info/, accessed on 10/03/2025).

Based on the affected cell types and the pattern of retinal degeneration, IRDs are classified into subtypes such as rod-cone dystrophy or retinitis pigmentosa (RP), cone-rod dystrophy (CRD), and macular dystrophy.

Despite significant advancements in next-generation sequencing (NGS) technologies, the underlying genetic cause remains unidentified in approximately 20% of IRD cases, suggesting the existence of novel genes and pathways or undetected pathogenic variants in previously recognized disease-associated genes.[2] Recently, mutations in adaptor protein complex 5 (AP-5) genes (*AP5Z1*, *AP5B1*, *AP5M1*) have been linked to retinal dystrophies, supporting discovery of novel genes in association with retinal degeneration with or without extra-retinal manifestations.[3] The AP-5-complex genes are essential for intracellular transport processes, particularly within late endosomes and lysosomes. Defects in these pathways can lead to multiorgan abnormalities and lysosomal storage diseases (LSDs).[4]

These impairments in lysosomal metabolic pathways result in lysosomal dysfunction and disrupt the degradation of cellular waste, leading to the accumulation of metabolic by-products and progressive cellular damages.[4] Patients with LSDs often present with multiorgan involvement, including abnormalities in central nervous system, developmental delays, and, in some cases, non-syndromic retinal degeneration.[4] To date, over 50 genes have been linked to LSDs, while three lysosomal genes have been specifically associated with non-syndromic retinal degeneration, including *MFSD8* (MIM: 611124), *HGSNAT* (MIM: 610453), and *CLN3* (MIM: 607042).[4–8]

AP-5 is critical for intracellular trafficking and lysosomal function particularly in organizing and transporting proteins within the late endosome-to-Golgi retrieval pathway. This process is important for cellular homeostasis and supporting proper lysosomal function.[9] Additionally, the AP-5 complex is believed to facilitate lysosome recovery from endolysosomes. Furthermore, the AP-5 complex consists of four subunits: beta, zeta, mu, and

[1]Department of Molecular and Human Genetics, Baylor College of Medicine, Houston, TX 77030, USA; [2]Department of Ophthalmology and Visual Sciences, Robert M. Brunson Center for Translational Vision Research, University of California, Irvine, Irvine, CA 92697, USA; [3]Department of Ophthalmology, Casey Eye Institute, Oregon Health & Science University, Portland, OR 97239, USA; [4]Watson Genetic Laboratory, North Kargar Street, Tehran, Iran; [5]Retina Foundation of the Southwest, Dallas, TX 75231, USA
[6]Lead contact
*Correspondence: ruic20@hs.uci.edu
https://doi.org/10.1016/j.xhgg.2026.100584.
© 2026 The Author(s). Published by Elsevier Inc. on behalf of American Society of Human Genetics.
This is an open access article under the CC BY license (http://creativecommons.org/licenses/by/4.0/).

sigma, encoded by the genes *AP5B1*, *AP5Z1* (MIM: 614368), *AP5M1*, and *AP5S1*, respectively.[10] Recently, the identification of bi-allelic variants in three AP-5 subunits laid a foundation for its essential role in macular functioning.[3]

In this study, we expand the description of bi-allelic variants in *AP5Z1* and *AP5B1* in five unrelated patients presenting with retinal dystrophy specifically macular degeneration. Our findings provide additional evidence that *AP5Z1* and *AP5B1* are critical for retinal function, and their deficiency leads to retinal degeneration, with or without extra-retinal phenotypes.

Material and methods

Study approval and design

This study was approved by the institutional review boards at Baylor College of Medicine (H-29697), University of California, Irvine (5702), and Oregon Health & Science University (IRB00002735). All individuals included in this study were clinically diagnosed with IRDs by expert ophthalmologists at Casey Eye Institute, Portland, OR, USA and Hazrat Rasul Akram Hospital, Tehran, Islamic Republic of Iran. Recruitment was carried out in accordance with the ethical principles outlined in the Declaration of Helsinki. Peripheral blood samples were collected from each participant following the provision of written informed consent. Genomic DNA was extracted using the QIAamp DNA Blood Mini Kit (Qiagen, Hilden, Germany) according to the manufacturer's protocol.

Genetic analysis

Unsolved IRD cases, previously subjected to clinical-panel-based genetic testing, underwent whole-genome and -exome sequencing (WGS and WES) using genomic DNA extracted at Baylor College of Medicine, Houston, TX, USA. As previously detailed, sequence alignment, variant calling, and downstream filtering were carried out by the Functional Genomics Core at Baylor College of Medicine.[2,11] Initial analysis was performed for the variants in known IRD genes using a list of genes present at RetNet (https://retnet.org/).

Predicted loss-of-function (LoF) variants, including nonsense, frameshift, and canonical splice site mutations, were prioritized. Missense variants were further evaluated based on evolutionary conservation and computational predictions. The functional impact of non-synonymous variants was predicted using REVEL v1.3,[12] and splicing effects of synonymous, non-synonymous, and intronic variants were assessed with SpliceAI v1.2.1.[13] Sequencing reads were aligned to the human reference genome (hg19) using the Burrows-Wheeler aligner (BWA),[14] and single nucleotide variants (SNVs) and insertion-deletions (INDELs) were identified using GATK4. Additionally, structural variant (SV) analysis, including copy number variations (CNVs) of WGS data for all of our unsolved patients, was performed during data processing. For the SV calling, we used Delly,[15] Lumpy,[16] Manta,[17] and CNVnator,[18] followed by annotation with AnnotSV.[19]

To exclude common variants unlikely to be disease causing, a population allele frequency (AF) threshold of 0.5% was applied. Coding-region variants were annotated using ANNOVAR and compared against the dbNSFP v3.5a database. Evolutionary conservation was assessed using phastCons scores from the University of California, Santa Cruz (UCSC) Genome Browser's 100-way alignment (phastCons.hg19.100way).[20] Variants that passed filtering were interpreted according to the American College of Medical Genetics and Genomics (ACMG) guidelines.[21] All prioritized variants were subsequently validated by Sanger sequencing.

Results

Clinical characteristics of the affected subjects

Participant P1 was a 74-year-old female of European (non-Finnish) descent who first experienced visual symptoms at age 50 years, including decreased visual acuity and photoaversion. Moreover, nyctalopia developed in her 60s, consistent with progressive retinal dysfunction (Figure 1A; Table 1).

Her family history includes a deceased brother with a muscular condition resembling amyotrophic lateral sclerosis (ALS) with no noticeable vision issues. Additionally, a deceased sister was diagnosed with macular degeneration prior to passing from lung cancer. Her niece was diagnosed with macular degeneration at age 45 years (Figure 1A).

At 71 years of age, best-corrected visual acuity (BCVA) was 20/40 in the right eye and 20/50 in the left eye. Refractive error was $0.50 + 2.00 \times 016°$ diopter (D) in right eye and $-0.25 + 1.50 \times 158°$ D in the left eye. Color-vision testing with Hardy-Rand-Rittler (HRR) pseudoisochromatic plates revealed severely reduced color vision in the right eye 0/6 and moderately reduced in the left eye 10/20 (Table 1). Goldmann kinetic visual field testing at the age of 71 years revealed significant bilateral visual field impairment and more severely damaged in the left eye. There were bilateral central and paracentral scotomas with reduced sensitivity in the mid-peripheral region. Kinetic visual field (KVF) images suggested progressive retinal degeneration from central to peripheral (Figure S1A).

Multimodal retinal imaging showed extensive bilateral macular atrophy with features consistent with advanced degenerative disease. Fundus photos and autofluorescence revealed central RPE loss and geographic atrophy, while optical coherence tomography (OCT) confirmed outer retinal thinning and ellipsoid zone disruption. The presence of outer retinal tubulations suggests a chorioretinal atrophy where the primary pathology originates in the RPE layer. These findings aligned with central and paracentral scotomas seen on perimetry (Figure 2). Full-field electroretinograms (ffERGs) demonstrated a significant reduction in both scotopic and photopic responses indicating widespread retinal rod and cone dysfunction (Figure S2A).

Participant P2 was a 65-year-old male of European (non-Finnish) descent and was diagnosed at age of 56 years with an atypical cone dystrophy. His visual symptoms began at 50 years with central scotomas. His medical history is

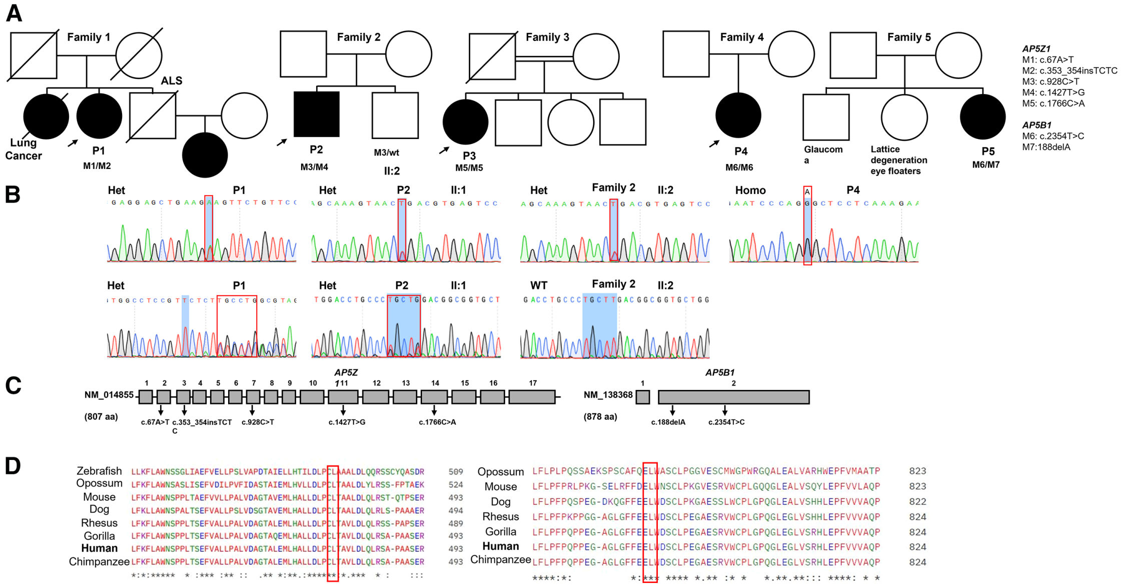

Figure 1. The family trees of the patients affected with AP5-complex-related retinopathy, Sanger sequencing, schematic representation of the genetic variations on the gene's structure, and multiple sequence alignment of AP5Z1 and AP5B1 proteins

(A) Pedigrees of the affected patients.
(B) Sanger sequencing of patients P1, P2, P4, and healthy brother of P2 (II:2).
(C) Schematic location 20 of the identified variants of *AP5Z1* and *AP5B1*. The exons are indicated by numbered boxes and introns are shown by horizontal lines; UTRs are not shown. The number of amino acids (aa) depicts protein size.
(D) Multiple sequence alignment of AP5Z1 and AP5B1 with their orthologs across different species at the sites of substitutions in AP5Z1 and AP5B1.

notable for syndactyly of the toes and a peripheral neuropathy affecting both hands and lower extremities. He also has a history of transient ischemic attack (TIA) along with systemic autoimmune and inflammatory conditions, including ankylosing spondylitis, gout, and lupus (Figure 1A and Table 1).

Family history was negative for CRD or hereditary spastic paraplegia. One brother has age-related vitreous detachment, but no other relatives had similar visual or neurological conditions (Figure 1A).

At the age of 61 years, BCVA was 20/70 in the right eye and counting fingers at 18 inches in the left eye. Refractive error in the right eye was $-6.00 + 0.75 \times 078°$ D and in the left eye was $-5.50 + 0.50 \times 117°$ D. Color-vision testing using HRR pseudoisochromatic plates showed significantly reduced chromatic discrimination (right eye 5/20; left eye 0/20) (Table 1).

At a follow-up examination at age 65 years, BCVA had measured 20/80 OD and 20/250 OS. Refractive error at that time was right eye $-4.50 + 0.75 \times 048°$ D and in left eye $5.00 + 0.50 \times 14°$ D (Table 1).

Additionally, KVF testing at the age of 56 and 60 years showed progressive dysfunction of macular region expanding peripherally (Table 1 and Figures S1B and S1C).

Multimodal retinal imaging at 61 and 65 years of age revealed extensive bilateral macular atrophy consistent with advanced degenerative retinal disease. OCT confirmed outer retinal thinning and disruption of the ellipsoid zone. The presence of outer retinal tubulations suggest chorioretinal atrophy where the primary pathology starts in the RPE. These findings correlate with the central and paracentral scotomas observed on perimetry (Figures 2 and S3). The ffERG for P2 demonstrates a significant bilateral reduction in both scotopic and photopic responses, indicating widespread retinal rod and cone dysfunction. (Figure S2C).

Participant P3, a 64-year-old Iranian female, presented with blurred vision, memory impairment, and hand tremors. Visual symptoms began at age 60 years, and there is no family history of similar vision or neurological conditions (Figure 1A and Table 1). Brain MRI revealed an intracranial meningioma and marked white matter leukoaraiosis, suggesting chronic small vessel ischemic changes that was a potential contribution to the neurological disease. At age 64 years, Humphrey visual field (30-2 SITA-Fast) testing demonstrated bilateral central scotomas, more pronounced in the right eye. The Visual Field Index (VFI) was 41% in the right eye and 52% in the left. This pattern of central visual-field loss with peripheral sparing was consistent with macular dystrophy (Figure S4).

Multimodal retinal imaging revealed bilateral structural and vascular abnormalities. Color fundus photographs demonstrated extensive vascular attenuation, telangiectasia, and possible intraretinal exudation. Fundus autofluorescence (FAF) imaging revealed a spotted pattern of hypo- and hyper-autofluorescence in the macular region of both eyes, indicating widespread RPE dysfunction and

Table 1. Demographic and clinical information of patients affected with *AP5*-related retinopathy

	P1	P2	P3	P4	P5
**Sex**	female	male	female	female	female
**Age at examination, (years)**	74	65	60	63	44
**Age of onset, (years)**	50	56	–	33	39
**Ethnicity**	European (non-Finnish)	European (non-Finnish)	Iran	European (non-Finnish)	European (non-Finnish)
**Gene**	*AP5Z1*	*AP5Z1*	*AP5Z1*	*AP5B1*	*AP5B1*
**Genomic variant (GRCh37)**	chr7-4820831 -A-T/chr7-4821372 -T- TTCTC	chr7-4824676-C-T/chr7-4827380-T-G	chr7-4829521-C-A/chr7-4829521-C-A	chr11-65545610-A-G	chr11-65545610-A-G/ chr11-65547775-CT-C
**HGVS annotation**	NM_014855(AP5Z1): c.67A>T(p.Lys 23Ter)/ NM_014855(AP5Z1): c.353_354insTCTC: (p.Leu120SerfsTer6)	NM_014855(AP5Z1):c.928C>T (p.Arg310Ter)/NM_014855(AP5Z1): c.1427T>G(p.Leu476Trp)	NM_014855(AP5Z1): c.1766C>A (p.Ser589Ter)/NM_ 014855(AP5Z1): c.1766C>A (p.Ser589Ter)	NM_138368(AP5B1):c.2354T>C (p.Leu785Pro)	NM_138368(AP5B1): c.2354T>C(p.Leu785Pro)/ NM_138368(AP5B1): c.188delA (p.Gln63ArgfsTer95)
**ACMG classification**	PVS1, PM2, PP5, (Pathogenic)/PVS1, PM2, PM3, (Pathogenic)	PM3, PVS1, PM2 (Pathogenic)/ PM2, PP3 (VUS)	PVS1, PM2 (Likely Pathogenic)	PM2, PP3 (VUS)	PM2, PP3 (VUS)/PVS1, PM2 (Likely Pathogenic)
**gnomAD v4.1.0 AF**	0.000001859/0	0.0001378/0	0	0.0006312	0.0006312/0.00004542
**gnomAD v4.1.0 subpopulation AF**	European (non-Finnish) 0.000001696, European (Finnish) 0/0	European (non-Finnish) 0.0001643, European (Finnish) 0/0	0	European (non-Finnish) 0.0007204, European (Finnish) 0.00009540	European (non-Finnish) 0.0007204, European (Finnish) 0.0007204/ 0.00005759
**Zygosity**	compound heterozygous	compound heterozygous	homozygous	homozygous	compound heterozygous
**Clinical diagnosis**	*AP5Z1*-related retinopathy	*AP5Z1*-related retinopathy	*AP5Z1*-related retinopathy	*AP5B1*-related retinopathy	*AP5B1*-related retinopathy
**Consanguinity**	no	no	yes	no	no
**Color vision**	RE: 0/6<br>LE: 10/20	RE: 5/20<br>LE: 0/20	N/A	RE:7/20<br>LE:4/20	no
**Visual acuity**	RE: 20/40<br>LE: 20/50	RE: 20/70<br>LE: CF	RE: 20/80<br>LE: 20/250	N/A N/A	RE: 20/70<br>LE: 20/400 N/A
**Refraction**	RE: −0.50 + 2.00x16°<br>LE: −0.25 + 1.50x158°	RE: −6.00 + 0.75x78°<br>LE: −5.50 + 0.50x117°	RE: −4.50 + 0.75x48°<br>LE: −5.00 + 0.50x141°	N/A	N/A N/A

(*Continued on next page*)

Table 1. *Continued*

	P1	P2	P3	P4	P5
**Fundus findings**	clear media, peripapillary atrophy, normal disk, normal vessels, macular atrophy in left eye, bilateral atrophic and pigmentary patches in posterior pole and midperiphery	prominent media opacity in the left>right eye. RPE atrophy, pigment mottling involving the macula and majority of posterior pole as well as mild vascular attenuation in both eyes. Mild changes centrally with increased atrophic area in both eyes	widespread RPE and chorioretinal atrophy	clear media in both eyes. Optic disk has large areas of peripapillary atrophy. The arterioles are mildly attenuated. The macula has large areas of atrophy with more pigmented clumps. Small central island in right eye with progression. There are reticular-like pigment changes in both eyes but the mid-peripheral retina are stable	clear media in both eyes. Optic disks showed peripapillary atrophy in both eyes with normal retinal vasculature. Macula exhibited pigment mottling with oval areas of RPE atrophy bilaterally. Peripheral retina demonstrated reticular pigmentary changes in both eyes
**Autofluorescence (FAF)**	foveal, perifoveal in left eye extrafoveal punched-out lesions in both eyes surrounding the central lesion in left eye and diffuse mottled in both eyes	patches of intense hypo-AF involving the macula as well as generalized hypo-autofluorescence of the posterior pole. Evaluation of the FAF of the left eye was limited by the media opacity before it was densely hypo-autofluorescence regions throughout the posterior poles, with intervening regions of hyper-AF in both eyes	well-demarcated areas are present in the posterior pole and midperiphery. These patches suggest bilateral extensive RPE atrophy. Surrounding areas show mixed granular hyper-autofluorescence, consistent with stressed or degenerating RPE cells	mild progression centrally in right eye, stable centrally in left eye and minimal changes peripherally in both eyes	oval areas of hypo-autofluorescence with some interval progression, reticular hyper-autofluorescence throughout the posterior pole in both eyes

(*Continued on next page*)

Table 1. *Continued*

	P1	P2	P3	P4	P5
**OCT**	showed severe outer retinal atrophy in both eyes with relative foveal sparing in right eye. Tubulations in both eyes. RNFL mapping showed irregular segmentation in both eyes	OCT demonstrated generalized loss of the outer retinal layers and RPE in the central macula with increased signal transitivity to the choroid and prominent outer retinal tubulations. Parafoveally, there is some preservation of the outer nuclear layer (ONL) in both eyes as well as focal preservation of the RPE in the right eye. The peripapillary and temporal macula have preservation of outer retinal structures previous visits showed outer segment attenuation, tubulations, subretinal deposits in both eyes. Islands of preserved outer segment structures persist centrally in both eyes	both eyes demonstrate marked macular thinning with outer retinal and RPE atrophy	large confluent patchy areas of severe outer retinal atrophy in the macula with outer retinal tubulations at the transition's zones of both eyes. There is residual preservation of the ONL at the fovea in right eye but not in left eye, which has mildly progressed. Choroidal thinning in the areas of atrophy in both eyes	areas of outer retinal atrophy with corresponding tubulations in both eyes
**Multifocal ERG**	N/A	severely decreased responses bilaterally. The R1 amplitude was 8.8 nv/deg^2 in right eye and 8.7 nv/deg^2 in left eye. These results are consistent with severe dysfunction of central macular cones in both eyes	N/A	the local first-order response P1 amplitude arrays (scalar-product) and ring averages were decreased left eye was worse than the right eye. The P1 implicit times were prolonged bilaterally and there was evidence of eccentric fixation in the right eye. The amplitude of the central hexagon of the right eye was 57 nv/deg2 and 20 nv/deg2 in left eye so, photopic cone ERG responses demonstrated subnormal amplitudes and prolonged implicit times. Thus, this multifocal ERG showed evidence of regional abnormality of macular cone responses in the pattern of a Bull's eye maculopathy	N/A

(*Continued on next page*)

Table 1. *Continued*

	P1	P2	P3	P4	P5
**ffERG**	showed a significant reduction in both scotopic and photopic responses, indicating widespread retinal dysfunction. The scotopic responses (DA 0.01, DA 3.0, and DA 10.0) showed reduced a- and b-wave amplitudes bilaterally, suggesting dysfunctional rod and bipolar cells. Photopic responses (LA 3.0) are also markedly reduced in both eyes, with low a- and b-wave amplitudes, consistent with cone cells dysfunctioning. Additionally, the 30 Hz flicker responses show low amplitude and delayed peaks relative to normal values, reflecting impaired cone-mediated temporal processing	ffER demonstrated a significant bilateral reduction in both scotopic and photopic responses, indicating widespread retinal dysfunction. Scotopic responses to dim and bright white flashes show markedly reduced a- and b-waves, reflecting impaired rod system activity. Both scotopic and photopic amplitudes are reduced, suggesting inner retinal dysfunction. Photopic single flash responses are similarly reduced, indicating cone system impairment, while the 30.3 Hz flicker responses show low amplitude and poor waveform, consistent with abnormal cone-mediated temporal processing. Overall, these findings are like the characteristics of widespread retinal dystrophy	N/A	dim scotopic responses showed normal amplitude and prolonged timing bilaterally (limited by blink) bright scotopic responses showed mildly reduced with normal timing bilaterally (limited by blink) dark-adapted cone responses showed normal amplitude and prolonged timing OU (limited by blink) single flash photopic responses showed normal amplitude and timing bilaterally photopic 30-Hz flicker showed mildly reduced amplitude and normal timing bilaterally measurements were limited by blink responses. These responses demonstrate mild reduction in 30-Hz flicker and prolonged timing in dim scotopic and dark-adapted cone responses. This is consistent with mild generalized cone dysfunction that reflects the severe macular dysfunction seen on the mfERG	ffERG showed normal amplitudes and normal implicit times of the rod-dependent responses. On the other hand, cone dependent responses showed normal amplitudes but abnormal implicit times indicating generalized abnormal retinal function of mild cone dysfunction
**Other symptoms**	no	syndactyly of toes of both feet, hearing loss, peripheral neuropathy	right frontal parafalx middle mass in brain	mild cataracts bilaterally	no

RE, right eye; LE, left eye; N/A, data not available.

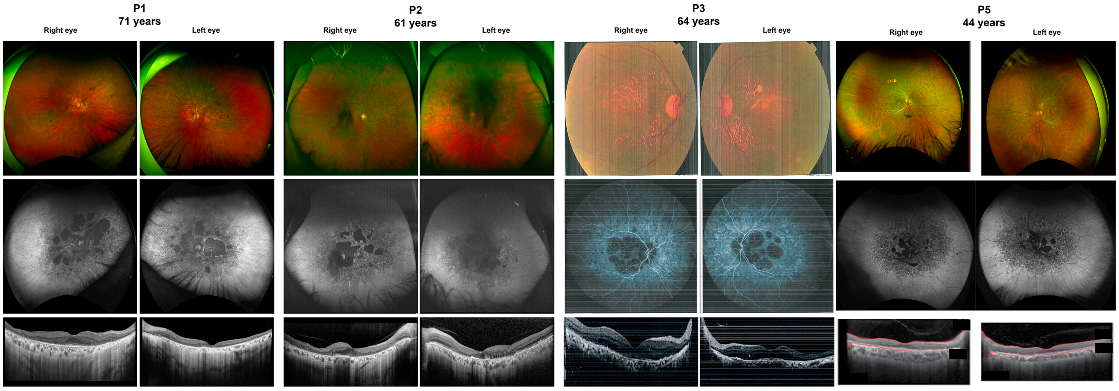

Figure 2. Multimodal retinal imaging of patients carrying *AP5Z1* and *AP5B1* bi-allelic variants
The top row displays multicolor or pseudocolor fundus images, the middle row shows fundus autofluorescence (FAF) or fluorescein angiography (FA) images, and the bottom row presents optical coherence tomography (OCT) images in each panel.
Web Resources https://retigene.erdc.info/, https://retnet.org/.

degeneration. Furthermore, OCT imaging confirmed severe disruption of the outer retinal layers, including loss of the ellipsoid zone and thinning of the photoreceptor layer, with localized subretinal deposits as well as RPE abnormalities (Figure 2).

Participant P4 was a 63-year-old female of European (non-Finnish) origin who was first diagnosed with retinal dystrophy at the age of 33 years. There was no family history of retinal disease (Figure 1A and Table 1).

At the age of 51 years, BCVA was 20/25 in the right eye and 20/50 in the left eye. Refractive error was $-5.75 + 1.00 \times 157°$ D in right eye and $-6.50 + 1.50 \times 007°$ D in left eye. Color-vision testing using the HRR pseudoisochromatic plates revealed reduced chromatic discrimination, with 7/20 in right eye and 4/20 in left eye. On follow-up examination at the age of 60 years, her visual function had significantly worsened, with BCVA declining to 20/70 OD and 20/400 OS (Table 1). KVF testing performed at ages 51 and 60 years demonstrated progressive expansion of the central scotomas in both eyes. (Figures S1D and S1E).

Multimodal imaging revealed progressive bilateral macular atrophy with extensive RPE loss and outer retinal degeneration. Wide-field fundus photographs show central pigmentation, RPE atrophy with visible choroidal vessels. FAF demonstrated large hypo-autofluorescent areas with surrounding hyper-autofluorescence. OCT showed outer retinal thinning and ellipsoid zone disruption.

Participant P5 was a 44-year-old European (non-Finnish) female (Figure 1A). Her symptoms started at the age of 39 years. Her initial clinical diagnosis was Stargardt disease. Her brother was affected by glaucoma, and her sister had a history of lattice degeneration. Her BCVA at the age of 43 years was 20/70 in OD and 20/400 OS (Table 1). Additionally, KVF testing at ages 43 and 44 years demonstrated progressive expansion of the central scotomas in both eyes (Figure S1G). Dilated fundus examination revealed peripapillary atrophy, pigment mottling, and oval areas of RPE atrophy in the macula and reticular pigmentary changes peripherally. FAF demonstrated oval hypo-autofluorescent areas and reticular hyper-autofluorescence throughout the posterior pole (Figures 2 and S3). OCT revealed outer retinal atrophy with corresponding tubulations suggesting primary loss of RPE. ffERG showed normal rod responses, whereas cone responses had normal amplitudes but delayed implicit times, indicating mild generalized cone dysfunction (Figure S2C).

Genetic findings

In this study, we identified bi-allelic variants in *AP5Z1* and *AP5B1* in five unrelated probands from US and Iranian cohorts who underwent WES or WGS after prior screening for known IRD genes. All patients exhibited progressive macular degeneration with chorioretinal atrophy as well as more variable generalized retinal dysfunction (Table 1).

A European patient, P1, was found to harbor very rare compound LoF variants in the *AP5Z1* gene. The first heterozygous variant, NM_014855 (AP5Z1):c.67A>T, introduces a premature stop codon at position 23 (p.Lys23Ter), while the second heterozygous variant NM_014855 (AP5Z1):c.353_354dupTCTC (p.Leu120SerfsTer6) results in a frameshift mutation because of the insertion of four nucleotides, predicted to cause an early stop codon at position 125 (Figures 1C and S5). These truncating variants likely lead to nonsense-mediated decay (NMD) of mRNA, ultimately resulting in complete loss of AP5Z1. Sanger sequencing confirmed both variants are heterozygous in this patient (Figure 1B). According to gnomAD v4.1.0, the global AFs of the NM_014855 (AP5Z1):c.67A>T (p.Lys23Ter) and NM_014855 (AP5Z1):c.353_354dupTCTC (p.Leu120SerfsTer6) variants are 0.000001859 and 0, respectively. Subpopulation-specific analysis revealed that c.67A>T (p.Lys23Ter) has a very low AF of 0.000001696 in the non-Finnish European population, and a frameshift variant NM_014855(AP5Z1):c.353_354dupTCTC (p.Leu 120SerfsTer6) was absent, revealing the identified variants are very rare (Table 1). These findings support the

pathogenic potential of the compound heterozygous variants in association with the observed phenotype in this patient.

Another European (non-Finnish) patient (P2) was found to carry rare compound heterozygous variants in *AP5Z1*. The first, NM_014855(AP5Z1):c.928C>T (p.Arg310Ter), introduces a premature stop codon predicted to trigger nonsense-mediated mRNA decay (NMD), thereby resulting in loss of gene function (Figures 1B and 1C). This LoF variant is rare, with a global AF of 0.0001378 in gnomAD v4.1.0 and a slightly higher frequency of 0.0001643 in non-Finnish Europeans. The second variant, NM_014855 (AP5Z1):c.1427T>G (p.Leu476Trp), is an extremely rare missense change absent from gnomAD v4.1.0 (Figure 1B and Table 1). Segregation analysis showed that the unaffected brother (II:2) is heterozygous for the NM_014855 (AP5Z1):c.928C>T:(p.Arg310Ter) variant, consistent with recessive inheritance (Figure 1B). The NM_014855 (AP5Z1):c.1427T>G (p.Leu476Trp) substitution has a high Combined Annotation Dependent Depletion (CADD) score of 24.6 and an AlphaMissense score of 0.7626, both supporting deleteriousness (Table S1). Furthermore, the affected residue is highly conserved across species (Figure 1D). Together, the presence of a protein-truncating allele and a rare predicted damaging missense allele strongly supports their contribution to the patient's phenotype.

Patient P3 is of Iranian origin and was identified to carry a homozygous LoF variant NM_014855 (AP5Z1): c.1766C>A (p.Ser589Ter) in *AP5Z1*, resulting in a premature stop codon at position p.Ser589Ter (Figures 1A and S5C). This variant is extremely rare, with heterozygote AF of 0.0000006446 in gnomAD v4.1.0 database, further supporting its potential pathogenicity. This nonsense variant is predicted to cause truncation of the AP5Z1 protein, likely leading to NMD of mRNA or, if translated, the production of a non-functional protein (Figure 1C).

A European (non-Finnish) patient (P4) was found to harbor homozygous missense variant NM_138368 (AP5B1):c.T2354T>C, resulting in a substitution of one amino acid leucine to proline at position 785 (p.Leu785-Pro) in the AP5B1 protein (Figure 1C). Sanger sequencing confirmed the identified variant is homozygous in this patient (Figure 1B). This variant is very rare in the general population, with a global AF of 0.0006312 in gnomAD and subpopulation-specific AF of 0.0007204 in the non-Finnish European. The variant is predicted to be deleterious, with a CADD score of 26.7 and AlphaMissense 0.707, suggesting a strong likelihood of pathogenicity (Table S1). Moreover, amino acid substitution occurs at position 785, which is highly conserved across different species (Figure 1D).

Interestingly, another unrelated European (non-Finnish) patient (P5) was found to carry the same NM_138368(AP5B1):c.T2354T>C (p.Leu785Pro) mutation along with compound heterozygous mutation NM_138368(AP5B1):c.188delA (p.Gln63ArgfsTer95) in *AP5B1* (Figure S6A and S6B). The heterozygous variant NM_138368(AP5B1):c.188delA (p.Gln63ArgfsTer95) is predicted to cause the early stop codon, hence resulting in no protein expression. This is a rare variant with a global AF 0.00004542 and subpopulation-specific AF 0.00005759 in non-Finnish Europeans (Table 1).

Discussion

In this study, we identified disease-causing variants in *AP5Z1* and *AP5B1* in five unrelated individuals with IRDs, providing additional evidence for the association between AP-5 complex genes and IRDs. All seven identified variants were classified as pathogenic, likely pathogenic, or variants of uncertain significance (VUSs) based on the 2015 ACMG/AMP criteria. Patients were evaluated at the Casey Eye Institute (Oregon) and in Iran. Clinically, all individuals presented with a late-onset macular degeneration progressing to chorioretinal atrophy and, in some cases, more generalized retinal dystrophy. The phenotype can overlap with late-onset ABCA4-related retinopathy but represents a distinct genetic entity. Based on our findings and from previous study, the available clinical data indicate a characteristic disease progression, beginning with early deposits (flecks), advancing to incomplete retinal atrophy at intermediate stages, and ultimately resulting in widespread chorioretinal atrophy extending from the macula to the peripheral retina. This pattern was observed during follow-up clinical visits in three patients from the previous study and four patients from our cohort, regardless of their genotype (Table 2). A shared feature of Stargardt disease, Stargardt-like diseases, pattern dystrophies, and central areolar choroidal dystrophy is the presence of fleck-like deposits at early and intermediate stages, reflecting RPE involvement in IRDs. Retinal features that differentiate these cases from ABCA4-associated retinopathy include late-onset development of macular and peri-macular well-demarcated hypo-autofluorescent lobular lesions that spare the fovea and are surrounded by regions of decreased autofluorescent and punctate areas of hyperautofluorescence. While these patients show similar features, their appearance overlaps with that seen in PRPH2-related retinopathy and ABCA4-related retinopathy, making it difficult to truly distinguish them based on appearance alone.[3]

Mutations in *AP5Z1* have previously been reported in the context of spastic paraplegia type 48 (SPG48) not specifically linked to retinal disease. Recent report, along with our findings, now describes retinal phenotypes in patients with *AP5Z1* variants.[3] Similarly, while *AP5B1* was only recently associated with retinal disease, our results provide independent confirmation of its role in IRDs.[3] We identified five variants in *AP5Z1* (four LoF and one missense) and two in *AP5B1* (one LoF and one missense), highlighting allelic heterogeneity in AP-5-related retinopathies. One of the identified variants NM_014855

Table 2. Phenotypic features of AP5-complex variants

ID	Gender	Variant 1	Variant 2	Age at examination (years)	Onset age (years)	Fundus	OCT	Other symptoms	Reference
**Previously reported patients**									
P1	M	NM_014855(AP5Z1):c.1836_1839dup(p.Leu614TyrfsTer150)	NM_014855(AP5Z1):c.1595G>T (p.Ser486_Arg532del)	63	50	central macular atrophy extending beyond temporal arcades, spared periphery, no peripapillary sparing. No optic disk pallor, only mildly attenuated vessels	cRORA and loss of choroid, ORT	N/A	Kaminska[3]
P2	M	NM_014855(AP5Z1):c.1836_1839dup(p.Leu614TyrfsTer150)	NM_014855(AP5Z1):c.1595G>T (p.Ser486_Arg532del)	74	40	central macular atrophy extending beyond temporal arcades, spared periphery, no peripapillary sparing. No optic disk pallor, only mildly attenuated vessels	cRORA and loss of choroid	N/A	Kaminska[3]
P3	M	NM_014855(AP5Z1):c.1836_1839dup(p.Leu614TyrfsTer150)	NM_014855(AP5Z1):c.1595G>T (p.Ser486_Arg532del)	63	40	central macular atrophy, spared periphery, no peripapillary sparing. No optic disk pallor, no attenuated vessels. Scarce peripheral reticular pigmentation on WF.	cRORA and loss of choriocapillaris, ORT	hearing loss	Kaminska[3]
P4	M	NM_014855(AP5Z1):c.1595G>T (p.Ser486_Arg532del)	NM_014855(AP5Z1):c.412C>T (p.Arg138Ter)	62	52	predominantly central atrophy, no peripapillary sparing. No optic disk pallor, no attenuated vessels. Reticular pigment deposition in the midperiphery on WF	cRORA and loss of choriocapillaris, ORT	paresthesia	Kaminska[3]
P5	M	NM_014855(AP5Z1):c.2086dup (p.Gln696ProfsTer67)	NM_014855(AP5Z1):c.2086dup (p.Gln696ProfsTer67)	43	43	perimacular atrophy, deposits extending beyond the temporal arcades. No optic disk pallor, no attenuated vessels	N/A	hearing loss	Kaminska[3]
P6	F	NM_014855(AP5Z1):c.950dup (p.Asp317GlufsTer93)	NM_014855(AP5Z1):c.1033C>T (p.Arg345Ter)	55	53	central atrophy with pigment; foveal sparing, spared periphery. No optic disk pallor	N/A	no	Kaminska[3]
P7	M	NM_014855(AP5Z1):c.1421_1447del(p.Pro474Leu482del)	NM_014855(AP5Z1):c.1421_1447del(p.Pro474_Leu482del)	56	47	central macular atrophy extending beyond temporal arcades and nasally, spared periphery, peripapillary area partially spared. No optic disk pallor, only mildly attenuated vessels	cRORA and loss of choriocapillaris, ORT	hearing loss, neurological	.Kaminska[3]
P8	M	NM_014855(AP5Z1):c.928C>T (p.Arg310Ter)	NM_014855(AP5Z1):c.928C>T (p.Arg310Ter)	63	45	macular chorioretinal atrophy with initial foveal sparing. Reticular pigment deposition in the periphery on WF	N/A	no	Kaminska[3]

(*Continued on next page*)

Table 2. *Continued*

ID	Gender	Variant 1	Variant 2	Age at examination (years)	Onset age (years)	Fundus	OCT	Other symptoms	Reference
P9	M	NM_014855(AP5Z1):c.928C>T (p.Arg310Ter)	NM_014855(AP5Z1):c.931C>T (p.Arg311Ter)	63	50	predominantly central atrophy, no peripapillary sparing. No optic disk pallor, no attenuated vessels. CHRPE in LE. Reticular pigment deposition in the periphery on WF	cRORA and loss of choriocapillaris, ORT	hearing loss,	Kaminska[3]
P10	M	NM_014855(AP5Z1):c.928C>T (p.Arg310Ter)	NM_014855(AP5Z1) deletion (7p22.1) including the AP5Z1 gene	65	45	central atrophy, spared periphery, no peripapillary sparing. No optic disk pallor, no attenuated vessels. Reticular pigment deposition in the periphery on WF	cRORA and loss of choroid and choriocapillaris, ORT	no	Kaminska[3]
P11	F	NM_014855(AP5Z1):c.928C>T (p.Arg310Ter)	NM_014855(AP5Z1) deletion (7p22.1) including the AP5Z1 gene	63	late 40s	central atrophy, extending beyond the temporal arcades, spared periphery, no peripapillary sparing. No optic disk pallor, no attenuated vessels. Reticular pigment deposition in the periphery on WF	cRORA and loss of choroid and choriocapillaris, ORT	hearing loss,	Kaminska[3]
P12	M	NM_014855(AP5Z1):c.1124_ 1132 + 75del (p.?)	NM_014855(AP5Z1):c.1124_ 1132 + 75del (p.?)	63	54	generalized atrophy with foveal sparing in RE. No optic disk pallor, no attenuated vessels	cRORA and loss of choriocapillaris	no	Kaminska[3]
P13	M	NM_014855(AP5Z1):c.412C>T (p.Arg138Ter)	NM_014855(AP5Z1):c.180_ 18G>A (p.?)	59	40	central macular atrophy	cRORA and loss of choriocapillaris, ORT	no	Kaminska[3]
P14	F	NM_014855(AP5Z1):c.80_ 83delinsTGCTGTAAAC TGTAACTGTAAA (p.Arg27delinsLeuLeuTer)	NM_014855(AP5Z1):c.1852dup (p.Leu618ProfsTer145)	32	31	yellow and pigmented deposits (macula and posterior pole including nasal to the optic disk)	no atrophy, outer retinal deposits	no	Kaminska[3]
P15	M	NM_014855(AP5Z1): c.824C>A(p.Ser275Ter)	NM_014855(AP5Z1):c.824C>A (p.Ser275Ter)	53	44	central and nasal atrophy, spared periphery, no peripapillary sparing. No optic disk pallor, no attenuated vessels. Reticular pigment deposition in the periphery	cRORA	Parkinsonism	Kaminska[3]
P16	F	NM_014855(AP5Z1):c.857_866del (p.Leu286ProfsTer25)	NM_014855(AP5Z1):c.857_866del (p.Leu286ProfsTer25)	52	mostly asymptomatic	flecks. No optic disk pallor, no attenuated vessels	N/A	spastic atactic paraparesis	Kaminska[3]
P17	F	NM_014855(AP5Z1): c.805C>T(p.Gln269Ter)	NM_014855(AP5Z1):c.805C>T (p.Gln269Ter)	44	42	macular yellow deposits. No optic disk pallor	N/A	N/A	Kaminska[3]

(*Continued on next page*)

Table 2. *Continued*

ID	Gender	Variant 1	Variant 2	Age at examination (years)	Onset age (years)	Fundus	OCT	Other symptoms	Reference
P18	M	NM_018229(AP5M1): c.97C>T (p.Arg33Ter)	NM_018229(AP5M1):c.97C>T (p.Arg33Ter)	46	30	asymmetric macular atrophy, yellow flecks. No optic disk pallor, no attenuated vessels. CHRPE in LE	outer retinal thickening in RE; outer retinal loss in LE, choroidal neovascular membrane, ORT	no	Kaminska[3]
P19	M	NM_018229.(AP5M1): c.1166G>A(p.Trp389Ter)	NM_018229(AP5M1): c.1166G>A(p.Trp389Ter)	59	57	predominantly central atrophy, relative foveal sparing, no peripapillary sparing. No optic disk pallor, no attenuated vessels	cRORA, ORT; choroidal thinning	Parkinsonism	Kaminska[3]
P20	M	NM_018229.(AP5M1): c.938A>G(p.Tyr313Cys)	NM_018229(AP5M1): c.938A>G(p.Tyr313Cys)	63	56	central atrophy, extending beyond the temporal arcades, spared periphery, no peripapillary sparing. No optic disk pallor, no attenuated vessels. Reticular pigment deposition in the periphery on WF	cRORA, ORT; choroidal thinning	no	Kaminska[3]
P21	M	NM_138368 (AP5B1): c.310del(p.Leu10 4TrpfsTer54)	NM_138368(AP5B1):c.310del (p.Leu104TrpfsTer54)	70	55	central atrophy, extending beyond the temporal arcades, spared periphery, no peripapillary sparing. No optic disk pallor, no attenuated vessels. Reticular pigment deposition in the periphery on WF	cRORA, ORT; choroidal thinning	N/A	Kaminska[3]
P22	F	NM_138368 (AP5B1): c.463C>T(p.Arg155Ter)	NM_138368(AP5B1):c.862del (p.Gln288SerfsTer29)	36	36	yellow flecks with gray halo at the posterior pole and periphery	subretinal deposits and thickening.	no	Kaminska[3]
**Patients in current study**									
P1	F	NM_014855(AP5Z1): c.67A>T(p.Lys 23Ter)	NM_014855(AP5Z1): c.353_354insTCTC (p.Leu120SerfsTer6)	74	50	clear media, peripapillary atrophy, normal disk, normal vessels, macular atrophy in LE, bilateral atrophic and pigmentary patches in posterior pole and midperiphery	cRORA and loss of choroid, ORT RNFL showed irregular segmentation in both eyes	no	this study
P2	M	NM_014855(AP5Z1): c.928C>T(p.Arg310Ter)	NM_014855(AP5Z1): c.1427T>G(p.Leu476Trp)	65	56	prominent media opacity in the left>right eye. RPE atrophy, pigment mottling involving the macula and majority of posterior pole as well as mild vascular attenuation in both eyes. Mild changes centrally with increased atrophic area in both eyes	cRORA and loss of choroid	syndactyly of toes of both feet, hearing loss, peripheral neuropathy	this study

(*Continued on next page*)

Table 2. *Continued*

ID	Gender	Variant 1	Variant 2	Age at examination (years)	Onset age (years)	Fundus	OCT	Other symptoms	Reference
P3	F	NM_014855(AP5Z1): c.1766C>A(p.Ser589Ter)	NM_014855(AP5Z1): c.1766C>(p.Ser589Ter)	60	N/A	widespread RPE and chorioretinal atrophy	cRORA and loss of choroid, ORT	brain abnormality	this study
P4	F	NM_138368(AP5B1): c.2354T>C(p.Leu785Pro)	NM_138368(AP5B1): c.2354T>C(p.Leu785Pro)	63	33	clear media in both eyes. Optic disk has large areas of peripapillary atrophy. The arterioles are mildly attenuated. The macula has large areas of atrophy with more pigmented clumps. Small central island in right eye with progression. There are reticular-like pigment changes in both eyes but the mid-peripheral retina are stable	cRORA and loss of choroid, ORT There is residual preservation of the ONL at the fovea in right eye but not in LE	mild cataracts bilaterally	this study
P5	F	NM_138368(AP5B1): c.2354T>C(p.Leu785Pro)	NM_138368(AP5B1): c.188delA(p.Gln6 3ArgfsTer95)	44	39	clear media in both eyes. Optic disks showed peripapillary atrophy in both eyes with normal retinal vasculature. Macula exhibited pigment mottling with oval areas of RPE atrophy bilaterally. Peripheral retina demonstrated reticular pigmentary changes in both eyes	cRORA and loss of choroid, ORT.	no	this study

CF, counting fingers; WF, wide-field fundus photography; cRORA, complete RPE and outer retinal atrophy; ORT, outer retinal tubulations; CHRPE, congenital hypertrophy of the retinal pigment epithelium; OCT, optical coherence tomography; N/A, not available; RNFL, retinal nerve fiber layer.

(*AP5Z1*):c.928C>T (p.Arg310Ter) has been associated with similar phenotype recently but other variants have never been associated with retinal dystrophies.[3] Moreover, two patients (P1 and P5) also had neurological symptoms. All patients carried bi-allelic variants: three were compound heterozygous and two homozygous. *In silico* predictions and conservation analyses supported pathogenicity, with LoF variants predicted to cause NMD or truncated proteins, and missense variants likely impairing protein structure, stability, or function.

While this manuscript was in preparation, another study described 23 AP-5-complex variants (mostly LoF) in 19 families with chorioretinal atrophy, predominantly of European ancestry.[3] In our study, four of our five patients are of European descent, suggesting that these specific IRDs are more frequent in European populations. Additionally, one variant, NM_014855 (*AP5Z1*):c.928C>T (p.Arg310Ter), identified in our patient was detected in three European families (British).[3] Additionally, two of our patients (P4 and P5) shared the same variant, NM_138368 (*AP5B1*):c.2354T>C (p.Leu785Pro), suggesting that these variants may have originated early and became more common in European populations. Notably, our case series also included one Iranian patient, supporting the need for analyses such as linkage disequilibrium decay to trace mutation origins across ethnic groups.

The AP-5 complex is essential for endolysosomal trafficking, and recent work has confirmed its functional importance in RPE cells. Despite relatively low retinal expression (Figures S6C, S6D, and S7), its conserved cellular role indicates a key function in retinal homeostasis. Interestingly, in our study, two patients carrying variants in *AP5Z1*, a gene previously associated with SPG48, also presented with neurological features. Patient P1 showed peripheral neuropathy and bilateral toe syndactyly, while patient P3 exhibited brain abnormalities. Similarly, previous studies have shown that patients carrying variants not only in *AP5Z1* but also in *AP5M1* and *AP5B1*—genes that had not previously been associated with any human phenotype—exhibit a broad spectrum of neurological features, including Parkinson disease, spastic ataxic paraparesis, intellectual disability, and polyneuropathy.[3] Defects in lysosomal pathways, such as those involving the AP-5 complex, can result in multiorgan abnormalities. Our findings therefore strengthen the genotype-phenotype correlation of AP-5-related disorders and support the inclusion of AP-5-complex genes in diagnostic panels for IRDs. Specifically, AP-5 complex should be considered in patients with a clinical diagnosis of Stargardt or Pattern dystrophy with prior negative genetic testing.

In conclusion, our study establishes variants in *AP5Z1* and *AP5B1* as autosomal-recessive causes of macular degeneration. Shared molecular pathways between these retinopathies, lysosomal disorders, and other retinal diseases suggest potential common mechanisms and therapeutic opportunities. Further *in vitro* and *in vivo* studies are needed to clarify disease mechanisms and explore targeted therapies. Our work highlights the value of WGS/WES in diagnosing unsolved IRDs and underscores the importance of considering AP-5 complex genes in patient management and counseling.

Data and code availability

- No large datasets or code were generated or analyzed in this study.

Acknowledgments

We are grateful to the patients and their families for their participation in this study. We also extend our thanks to Dr. Kim Carlyle Worley for her support. We also acknowledge funding support to the Gavin Herbert Eye Institute at the University of California, Irvine, provided by an unrestricted grant from Research to Prevent Blindness and the NIH core grant P30 EY034070.

The funding was provided by the National Eye Institute (EY022356, EY018571, EY002520, P30EY010572, and EY030 499), the Retinal Research Foundation, an NIH shared instrument grant (S10OD023469), the Daljit S. and Elaine Sarkaria Charitable Foundation, an unrestricted grant from Research to Prevent Blindness (New York), Knights Templar Eye Foundation grant 2024 #9, Fighting Blindness Canada, and the Vision Health Research Network. The funding agencies did not have any involvement in this study. This work was supported by the National Institutes of Health (Bethesda, MD) P30 EY010572 core grant, the Malcolm M. Marquis, MD Endowed Fund for Innovation, and an unrestricted grant from Research to Prevent Blindness (New York, NY) to Casey Eye Institute, Oregon Health & Science University (P.Y. and M.E.P.).

Author contributions

H.M.J.H., M.E.P., and R.C. designed the study. M.E.P., R.L.C., E.F.-O., P.Y., M.K., and M.A. collected clinical data. Y.L. and B.T. performed sequencing. H.M.J.H. and M.W. analyzed the sequencing data. H.M.J.H. drafted and M.E.P. and R.C. edited the manuscript. All authors revised the manuscript.

Declaration of interests

The authors declare no competing interests.

Supplemental information

Supplemental information can be found online at https://doi.org/10.1016/j.xhgg.2026.100584.

Received: December 1, 2025
Accepted: February 23, 2026

References

1. Schneider, N., Sundaresan, Y., Gopalakrishnan, P., Beryozkin, A., Hanany, M., Levanon, E.Y., Banin, E., Ben-Aroya, S., and Sharon, D. (2022). Inherited retinal diseases: Linking genes,

disease-causing variants, and relevant therapeutic modalities. Prog. Retin. Eye Res. *89*, 101029. https://doi.org/10.1016/j.preteyeres.2021.101029.

2. Hussain, H.M.J., Wang, M., Huang, A., Schmidt, R., Qian, X., Yang, P., Marra, M., Li, Y., Pennesi, M.E., and Chen, R. (2023). Novel Pathogenic Mutations Identified from Whole-Genome Sequencing in Unsolved Cases of Patients Affected with Inherited Retinal Diseases. Genes *14*, 447. https://doi.org/10.3390/genes14020447.
3. Kaminska, K., Cancellieri, F., Quinodoz, M., Moye, A.R., Bauwens, M., Lin, S., Janeschitz-Kriegl, L., Hayman, T., Barberán-Martínez, P., Schlaeger, R., et al. (2025). Bi-allelic variants in three genes encoding distinct subunits of the vesicular AP-5 complex cause hereditary macular dystrophy. Am. J. Hum. Genet. *112*, 808–828. https://doi.org/10.1016/j.ajhg.2025.02.015.
4. Platt, F.M., d'Azzo, A., Davidson, B.L., Neufeld, E.F., and Tifft, C.J. (2018). Lysosomal storage diseases. Nat. Rev. Dis. Primers *4*, 27. https://doi.org/10.1038/s41572-018-0025-4.
5. Wang, F., Wang, H., Tuan, H.F., Nguyen, D.H., Sun, V., Keser, V., Bowne, S.J., Sullivan, L.S., Luo, H., Zhao, L., et al. (2014). Next generation sequencing-based molecular diagnosis of retinitis pigmentosa: identification of a novel genotype-phenotype correlation and clinical refinements. Hum. Genet. *133*, 331–345. https://doi.org/10.1007/s00439-013-1381-5.
6. Haer-Wigman, L., Newman, H., Leibu, R., Bax, N.M., Baris, H.N., Rizel, L., Banin, E., Massarweh, A., Roosing, S., Lefeber, D.J., et al. (2015). Non-syndromic retinitis pigmentosa due to mutations in the mucopolysaccharidosis type IIIC gene, heparan-alpha-glucosaminide N-acetyltransferase (HGSNAT). Hum. Mol. Genet. *24*, 3742–3751. https://doi.org/10.1093/hmg/ddv118.
7. Roosing, S., van den Born, L.I., Sangermano, R., Banfi, S., Koenekoop, R.K., Zonneveld-Vrieling, M.N., Klaver, C.C.W., van Lith-Verhoeven, J.J.C., Cremers, F.P.M., den Hollander, A.I., and Hoyng, C.B. (2015). Mutations in MFSD8, encoding a lysosomal membrane protein, are associated with nonsyndromic autosomal recessive macular dystrophy. Ophthalmology *122*, 170–179. https://doi.org/10.1016/j.ophtha.2014.07.040.
8. Bauwens, M., Storch, S., Weisschuh, N., Ceuterick-de Groote, C., De Rycke, R., Guillemyn, B., De Jaegere, S., Coppieters, F., Van Coster, R., Leroy, B.P., and De Baere, E. (2020). Functional characterization of novel MFSD8 pathogenic variants anticipates neurological involvement in juvenile isolated maculopathy. Clin. Genet. *97*, 426–436. https://doi.org/10.1111/cge.13673.
9. Hirst, J., Itzhak, D.N., Antrobus, R., Borner, G.H.H., and Robinson, M.S. (2018). Role of the AP-5 adaptor protein complex in late endosome-to-Golgi retrieval. PLoS Biol. *16*, e2004411. https://doi.org/10.1371/journal.pbio.2004411.
10. Hirst, J., Hesketh, G.G., Gingras, A.C., and Robinson, M.S. (2021). Rag GTPases and phosphatidylinositol 3-phosphate mediate recruitment of the AP-5/SPG11/SPG15 complex. J. Cell Biol. *220*, e202002075. https://doi.org/10.1083/jcb.202002075.
11. Brock, D.C., Wang, M., Hussain, H.M.J., Rauch, D.E., Marra, M., Pennesi, M.E., Yang, P., Everett, L., Ajlan, R.S., Colbert, J., et al. (2024). Comparative analysis of in-silico tools in identifying pathogenic variants in dominant inherited retinal diseases. Hum. Mol. Genet. *33*, 945–957. https://doi.org/10.1093/hmg/ddae028.
12. Ioannidis, N.M., Rothstein, J.H., Pejaver, V., Middha, S., McDonnell, S.K., Baheti, S., Musolf, A., Li, Q., Holzinger, E., Karyadi, D., et al. (2016). REVEL: An Ensemble Method for Predicting the Pathogenicity of Rare Missense Variants. Am. J. Hum. Genet. *99*, 877–885. https://doi.org/10.1016/j.ajhg.2016.08.016.
13. Jaganathan, K., Kyriazopoulou Panagiotopoulou, S., McRae, J.F., Darbandi, S.F., Knowles, D., Li, Y.I., Kosmicki, J.A., Arbelaez, J., Cui, W., Schwartz, G.B., et al. (2019). Predicting Splicing from Primary Sequence with Deep Learning. Cell *176*, 535–548.e24. https://doi.org/10.1016/j.cell.2018.12.015.
14. Li, H., and Durbin, R. (2009). Fast and accurate short read alignment with Burrows-Wheeler transform. Bioinformatics *25*, 1754–1760. https://doi.org/10.1093/bioinformatics/btp324.
15. Rausch, T., Zichner, T., Schlattl, A., Stütz, A.M., Benes, V., and Korbel, J.O. (2012). DELLY: structural variant discovery by integrated paired-end and split-read analysis. Bioinformatics *28*, i333–i339. https://doi.org/10.1093/bioinformatics/bts378.
16. Layer, R.M., Chiang, C., Quinlan, A.R., and Hall, I.M. (2014). LUMPY: a probabilistic framework for structural variant discovery. Genome Biol. *15*, R84. https://doi.org/10.1186/gb-2014-15-6-r84.
17. Chen, X., Schulz-Trieglaff, O., Shaw, R., Barnes, B., Schlesinger, F., Källberg, M., Cox, A.J., Kruglyak, S., and Saunders, C.T. (2016). Manta: rapid detection of structural variants and indels for germline and cancer sequencing applications. Bioinformatics *32*, 1220–1222. https://doi.org/10.1093/bioinformatics/btv710.
18. Abyzov, A., Urban, A.E., Snyder, M., and Gerstein, M. (2011). CNVnator: an approach to discover, genotype, and characterize typical and atypical CNVs from family and population genome sequencing. Genome Res. *21*, 974–984. https://doi.org/10.1101/gr.114876.110.
19. Geoffroy, V., Herenger, Y., Kress, A., Stoetzel, C., Piton, A., Dollfus, H., and Muller, J. (2018). AnnotSV: an integrated tool for structural variations annotation. Bioinformatics *34*, 3572–3574. https://doi.org/10.1093/bioinformatics/bty304.
20. Pollard, K.S., Hubisz, M.J., Rosenbloom, K.R., and Siepel, A. (2010). Detection of nonneutral substitution rates on mammalian phylogenies. Genome Res. *20*, 110–121. https://doi.org/10.1101/gr.097857.109.
21. Richards, S., Aziz, N., Bale, S., Bick, D., Das, S., Gastier-Foster, J., Grody, W.W., Hegde, M., Lyon, E., Spector, E., et al. (2015). Standards and guidelines for the interpretation of sequence variants: a joint consensus recommendation of the American College of Medical Genetics and Genomics and the Association for Molecular Pathology. Genet. Med. *17*, 405–424. https://doi.org/10.1038/gim.2015.30.

HGGA, Volume 7

Supplemental information

Bi-allelic variants in *AP5Z1* and *AP5B1*

lead to retinal degeneration

Hafiz Muhammad Jafar Hussain, Meng Wang, Paul Yang, Behnoosh Tasharrofi, Yumei Li, Rebecca Lynn Clark, Emma Fale-Olsen, Grace Waldow, Mohammad Keramatipour, Mostafa Asadollahi, Mark E. Pennesi, and Rui Chen

**Table S1.** ***In-silico*** **prediction of identified variants**			
**Variant**	**Tool**	**Scores**	**Prediction**
NM_014855(AP5Z1):c.67A>T (p.Lys 23Ter)	CADD	35	deleterious
NM_014855(AP5Z1):c.928C>T (p.Arg310Ter)	CADD	45	deleterious
NM_014855: (*AP5Z1*):c.1427T>G (p.Leu476Trp)	REVEL	0.6	uncertain
	AlphaMissense	0.763	deleterious
	CADD	24.6	deleterious
	SIFT	0.001	uncertain
NM_014855(AP5Z1):c.1766C>A (p.Ser589Ter)	CADD	40	deleterious
NM_138368(*AP5B1*):c.2354T>C:(p.Leu 785Pro)	REVEL	NA	NA
	AlphaMissense	0.707	uncertain
	CADD	24.6	deleterious
	SIFT	0	deleterious
NA, not available.			

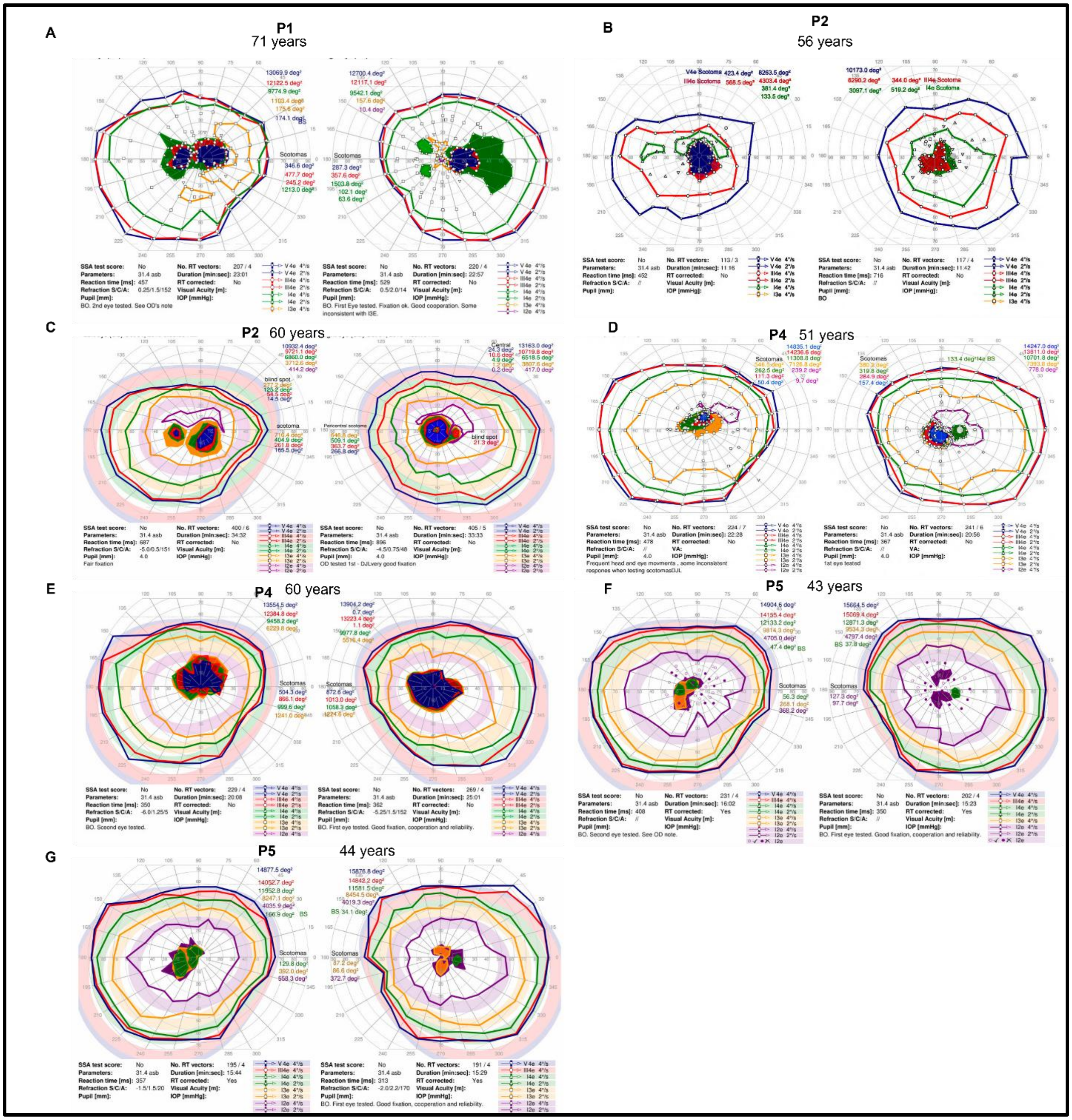

Figure S1. Kinetic Visual Fields (KVF) of P1, P2, P4 and P5. (A) KVF of patient P1 at the age of 71 years. (B) KVF of patient P2 at the age of 56 years. (C) KVF of patient P2 at the age of 60 years. (D) KVF of patient P4 at the age of 51 years. (E) KVF of patient P4 at the age of 60 years. (F) KVF of patient P5 at the age of 43 years. (G) KVF of patient P5 at the age of 44 years. All visual fields show central scotomas with preserved peripheral vision, and the findings demonstrate age-related progression, confirming chorioretinal atrophy in all patients.

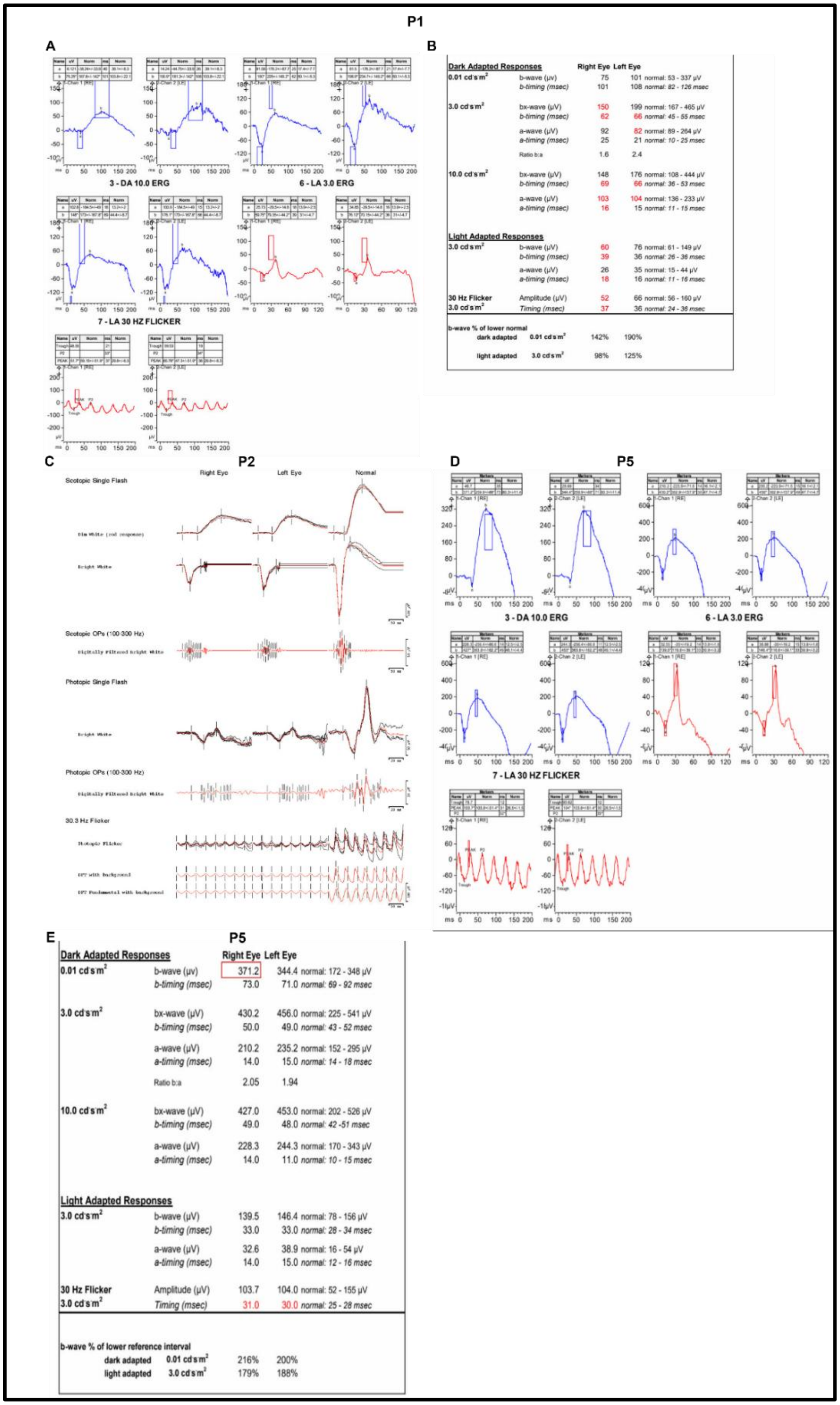

Figure S2. Full field electroretinography (ffERG) of patients P1 and P5. (A) ffERG of P1. The labeling follows standard conventions, indicating the eye's adaptive state [dark-

adapted (DA) or light-adapted (LA)] and the flash intensity (in cd·s/m²). This ERG was performed on an Espion E3 system that was calibrated to the ISCEV standards. (B) Peak amplitudes and timings for each stimulus for patient P1. These recordings demonstrate normal rod function but show mild cone dysfunction as indicated by borderline amplitudes and prolonged timings. Thes finding are consistent with macular dysfunction. (C) ffERG of P2. (D) ffERG of P5. (E) Peak amplitudes and timings for each stimulus for patient P5. These recordings demonstrate normal rod function but show mild cone dysfunction as indicated by prolonged timings of the 30Hz flicker responses.

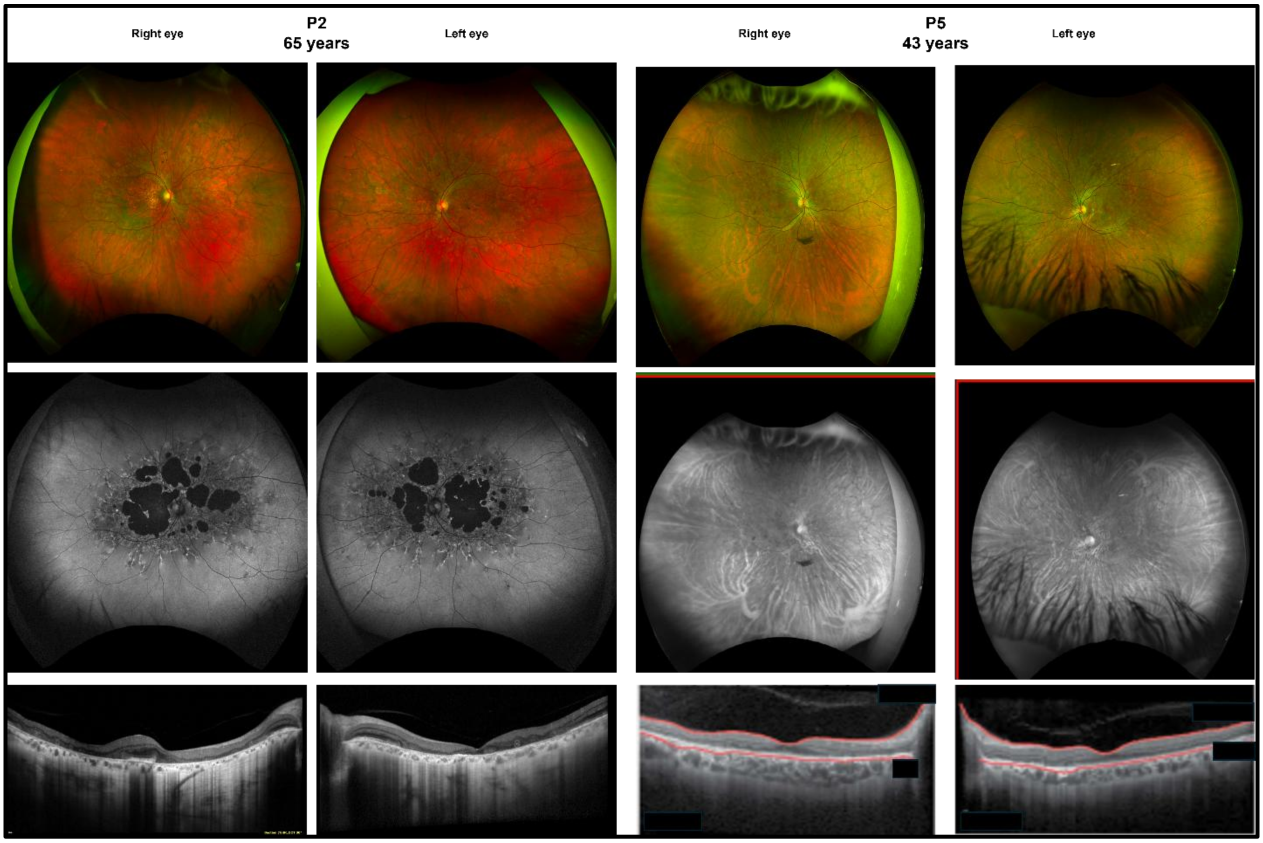

Figure S3. Multimodal retinal imaging of patients P2 and P5 carrying *AP5Z1* and *AP5B1* bi-allelic variants respectively. The top row displays multicolor or pseudocolor fundus images, the middle row shows fundus autofluorescence (FAF) images, and the bottom row presents optical coherence tomography (OCT) images in each panel.

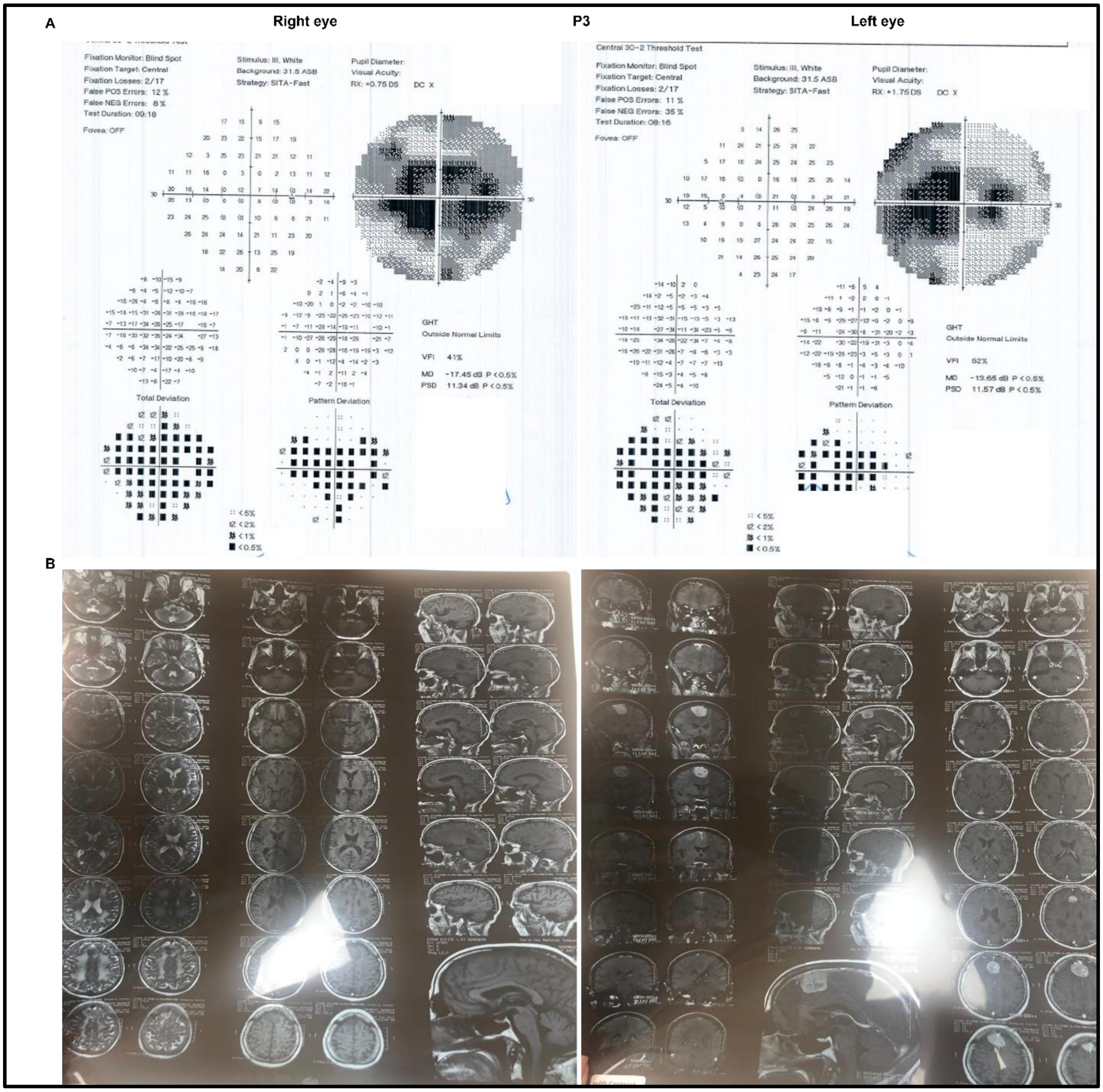

Figure S4. Humphrey Visual fields and brain MRI of patient P3. (A) Visual field test of patient P3. (B) Brain MRI of patient P3.

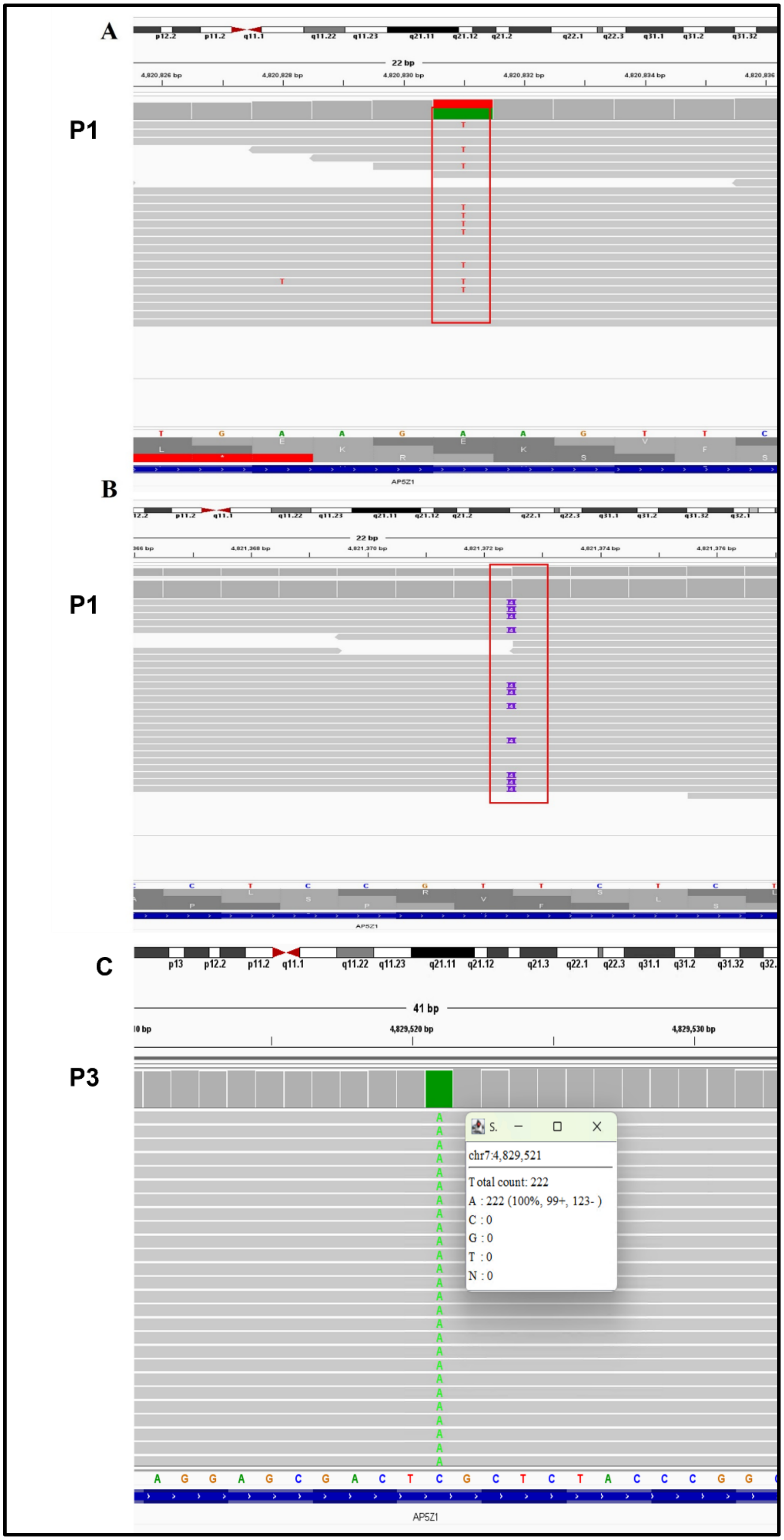
A
P1
22 bp
AP5Z1
B
P1
22 bp
AP5Z1
C
P3
41 bp
4,829,520 bp
4,829,530 bp
chr7:4,829,521
Total count: 222
A : 222 (100%, 99+, 123-)
C : 0
G : 0
T : 0
N : 0
AP5Z1

Figure S5. IGV and identified variant's location in P1 and P3. (A) IGV plot showing the variant region of patient (P1) affected with *AP5Z1*-related retinopathy had a likely pathogenic heterozygous stop-gain chr7:4820831-A-T. (B) IGV plot showing the variant region of patient (P1) affected with *AP5Z1*-related retinopathy had a likely pathogenic heterozygous insertion of four nucleotides chr7:4821372-T-TTCTC in *AP5Z1* gene. (C) IGV plot showing the variant region of patient (P3) affected with *AP5Z1*-related retinopathy had a likely pathogenic homozygous nonsense variant chr7-4829521-C-A in *AP5Z1* gene.

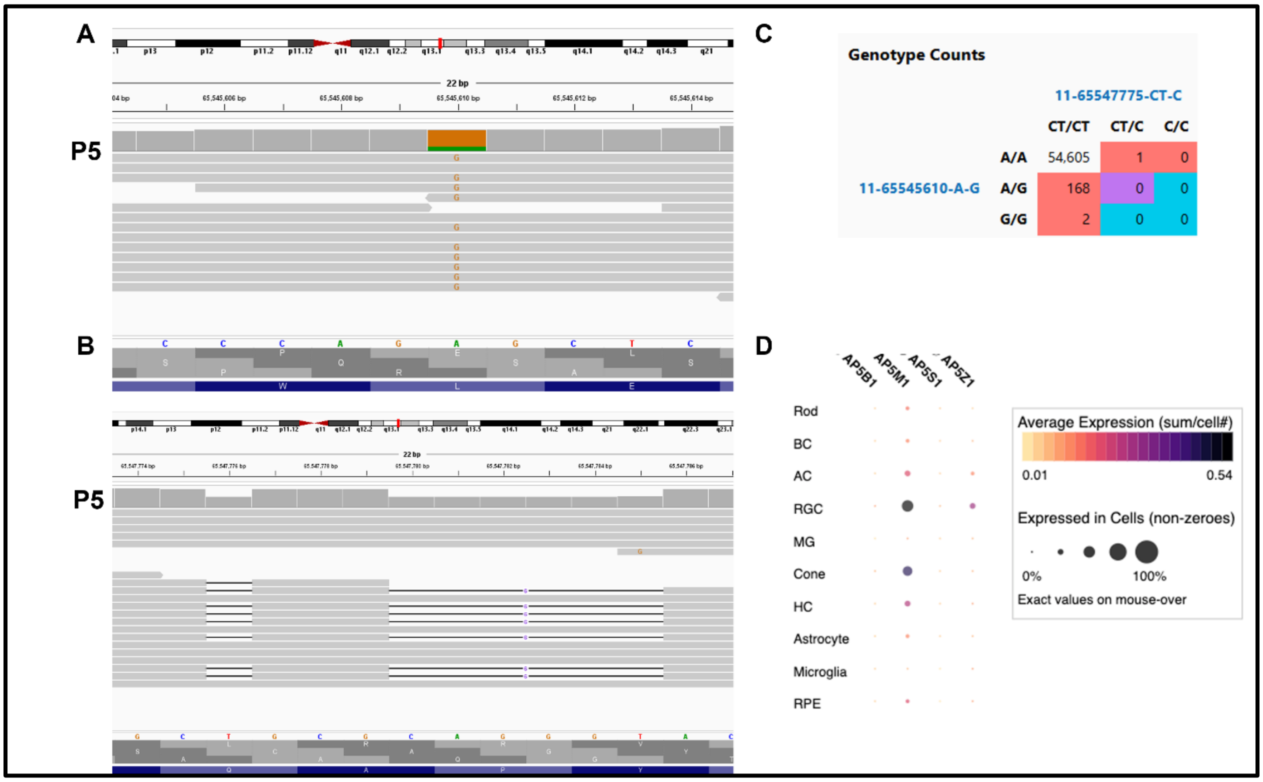

Figure S6. IGV of P5 and AP5 complex genes expression. (A) IGV plot showing the variant region of patient (P5) affected with *AP5B1*-related retinopathy had heterozygous missense variant chr11-65545610-A-G. (B) IGV plot showing the variant region of patient (P5) affected with *AP5B1*-related retinopathy had a heterozygous deletion of one nucleotide, resulting in stop codon chr11-65547775-CT-C. (C) Phasing analysis from gnomAD v2 of identified variants in patient P5 (chr11-65545610-A-G: (NM_138368) c.2354T>C:p.Leu785Pro/chr11-65547775-CT-C: c.188delA:p.Gln63Argfs*95. (D) Single nuclei expression of *AP5* complex genes in major retina cell classes.

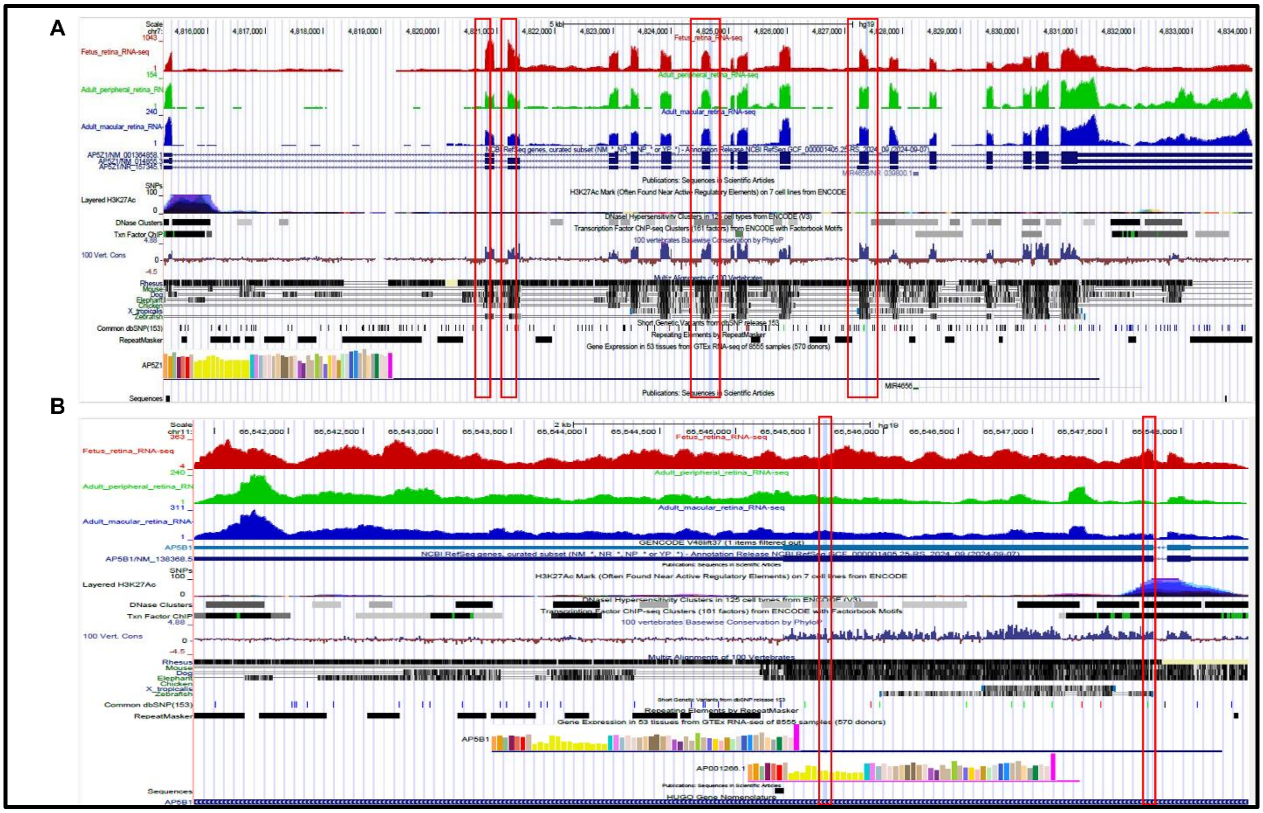

Figure S7. RNA-seq expression of AP5Z1 (A) and AP5B1 (B) in fetus and adult retina and location of identified variants in both genes (red enclosed).
